# Temporal stability of child growth associations in Demographic and Health Surveys in 25 countries

**DOI:** 10.1016/j.ssmph.2019.100352

**Published:** 2019-01-12

**Authors:** Matthias Rieger, Sofia Karina Trommlerová, Radu Ban, Kristen Jeffers, Matthew Hutmacher

**Affiliations:** a International Institute of Social Studies, Erasmus University Rotterdam, P.O. Box 29776, 2502 LT, The Hague, The Netherlands; b Universitat Pompeu Fabra, Plaça de la Mercè 10, 08002, Barcelona, Spain; c Bill & Melinda Gates Foundation, 500 Fifth Avenue North Seattle, WA 98109, USA; d University of Minnesota, Minnesota Population Center, 50 Willey Hall, 225 19th Avenue South, Minneapolis, MN 55455, USA; e Ann Arbor Pharmacometrics Group (Formerly), 900 Victors Way #328, Ann Arbor, MI 48108, USA

**Keywords:** DHS, Demographic and Health Survey, HAZ, height-for-age z-score, HBGDki, Healthy, Birth, Growth, and Development knowledge integration, WHO, World Health Organization, Temporal stability, Height-for-age, Stunting, Demographic and Health Surveys

## Abstract

**Background:**

Socio-economic and demographic determinants of child growth at ages 0–5 years in developing countries are well documented. However, Precision Public Health interventions and population targeting require more finely grained knowledge about the existence and character of temporal changes in child growth associations.

**Methods:**

We evaluated the temporal stability of associations between height-for-age z-score (HAZ) of children aged 0–59 months and child, parental, household, and community and infrastructure factors by following 25 countries over time (1991–2014) in repeated cross-sections of 91 Demographic and Health Surveys using random effect models and Wald tests.

**Results:**

We found that child growth displayed relatively more time stable associations with child, parental, and household factors than with community and infrastructure factors. Among the unstable associations, there was no uniform geographical pattern in terms of where they consistently increased or decreased over time. There were differences between countries in the extent of temporal instability but there was no apparent regional grouping or geographic pattern. The instability was positively and significantly correlated with annual changes in HAZ.

**Conclusions:**

These findings inform about the generalizability of results stemming from cross-sectional studies that do not consider time variation – results regarding effects of child, parental, and household factors on HAZ do not necessarily need to be re-evaluated over time whereas results regarding the effects of infrastructure and community variables need to be monitored more frequently as they are expected to change. In addition, the study may improve the Precision Public Health population targeting of interventions in different regions and times – whereas the temporal dimension seems to be important for precision targeting of community and infrastructure factors, it is not the case for child, parental, and household factors. In general, the existence of temporal instability and the direction of change varies across countries with no apparent regional pattern.

## Highlights

• Evaluates the temporal stability of determinants of height-for-age z-score (HAZ).
• Associations of HAZ with child, parental, and household factors are relatively stable.
• Associations with community and infrastructure factors are more unstable.
• Temporal instability was positively correlated with average annual changes in HAZ.
• Finds no apparent regional grouping or geographic pattern in temporal instability.

## 1 Introduction

In 2015, there were 156 million stunted under-5 children globally (UNICEF, 2016). Stunting in early childhood is a primary driver of mortality and has adverse effects on nutritional status, health, and socio-economic outcomes in later life (Black et al., 2008, Hoddinott et al., 2013, Hoddinott et al., 2008, Mendez and Adair, 1999, Glewwe and Miguel, 2007, Strauss and Thomas, 1998). Although the global percentage of stunted children has declined from 33% to 23% between 2000 and 2015, there is substantial regional heterogeneity (UNICEF, 2016).

This paper is part of the Healthy Birth, Growth, and Development knowledge integration initiative (HBGDki). The initiative was established by the Bill & Melinda Gates Foundation to engage the global health community by using shared data and modern analytics to generate new knowledge about preterm birth, growth faltering, and impaired neurocognitive development (Jumbe, Murray, & Kern, 2016). The goals of HBGDki include strategy development to solve stunting globally by delivering the right treatment to the right child at the right time, place, and cost, as opposed to a one-size-fits-all approach, which is unlikely to solve global stunting (HBGDki, 2018). This effort falls into the emerging field of *Precision Public Health,* which can be defined as the leveraging of predictive models and existing data to detect, identify, track, and treat traits of unique subpopulations with greater accuracy. The term can be traced back to (yet should not be confused with) *Precision Medicine*, which in turn tailors treatments to individual patients rather than sub-populations and geographies (see Desmond-Hellmann, 2016). For instance, while access to sanitation has been found to predict child growth on average, this may not be true in all geographies, sub-populations and time periods (Hathi, Haque, Pant, Coffey, & Spears, 2017). Identifying the right target groups requires models that allow for heterogeneity in space and time. In this sense, it is important to develop knowledge about temporal and spatial differences between and within regions as a foundation for targeted strategies and policies to reduce stunting globally.

This motivated the present study to examine the temporal stability of associations between height-for-age z-scores and demographic and socio-economic determinants of child malnutrition in developing countries such as birth order, sex, maternal height, parental education, and access to sanitation (Desai and Alva, 1998, Hathi et al., 2017, Özaltin et al., 2010; Thomas, Strauss, & Henriques, 1991). Previous studies using data from Bangladesh, India, Nepal, and Pakistan showed that the associations between child growth and socio-economic variables were stable over time (Headey et al., 2015, Headey et al., 2016). However, these studies did not focus primarily on temporal stability of these associations and they included only a few countries.^2^

The purpose of the present study was to evaluate changes in the magnitude and direction of the associations between different demographic and socio-economic variables and height-for-age z-score (HAZ) by following countries over time in repeated cross-sections. We aimed to identify the variables that had the most and least volatile associations with HAZ, and rank countries by the temporal instability of these associations. We then evaluated whether initial levels of HAZ and changes in HAZ may explain observed cross-country differences in instability of associations. We also tested whether documented instabilities followed a linear trend and if so, whether the trend differed between countries. Our analysis was of exploratory nature and we had no specific hypotheses regarding which variables would be more stable than others. Nevertheless, we did expect to find more stable associations with slow moving individual factors (such as maternal height or education) compared to more dynamic community or infrastructure variables (such as sanitation or electricity). Taken together, our results may inform child growth prediction efforts and modelling strategies using the widely employed DHS surveys.

## 2 Methods

### 2.1 Data sources and procedures

We used pooled Demographic and Health Surveys (DHS) made available by ICF International (2016). The DHS were nationally representative cross-sectional surveys performed in low and middle-income countries since the late 1980s. The surveys, accessed from the DHS Program and IPUMS-DHS, were harmonized for comparability across time and space (Heger Boyle, King, & Sobek, 2017). The data were freely accessible after online registration. Details pertaining to data collection procedures and data access were documented on the DHS website (dhsprogram.com). The DHS focused on health of women of reproductive age and their children using standard questionnaires (within DHS phase and subject to country-specific modifications) and survey procedures across countries. The DHS program aimed to implement surveys every five years in developing countries, but survey frequency depended on the requests and needs of countries and key donors such as United States Agency for International Development. All consenting women aged 15–49 years in sampled households were interviewed; in some samples only ever-married women were interviewed. Height and weight measures of these women’s surviving and co-resident children aged 0–59 months (in some surveys 0–35 or 0–47 months) were collected. Additional household information was collected from one household member.

There was no formal study protocol because the data were publicly available and the study was exploratory. For sample determination, we aimed to study the 39 priority countries identified by the Bill & Melinda Gates Foundation that previous work (Black et al., 2013) identified to bear for most of the global burden of stunting. For 25 of these countries, there were at least two DHS rounds with all covariates available, thus enabling testing for temporal stability. We used all survey rounds available at data curation in March 2016. We assembled 91 surveys with 689,529 under-5 children. There were 70,390 children who did not have valid anthropometric measures because of missing height measurement, implausible HAZ, or anthropometric measurements being administered only to under-3 or under-4 children in some surveys. In addition, 27,337 children were excluded from the analysis because explanatory variables were missing; 99.9% excluded children were due to missing maternal height or electricity (maternal height unavailable for 13,630 children, mainly due to Kenya 2014 DHS in which height was available for only half the mothers; electricity data missing for 13,451 children). The final analysis sample included 591,802 children in 25 countries from years 1991 to 2014. There were an average of 3.6 survey rounds per country (range, 2 [Chad, Democratic Republic of Congo, India] to 7 surveys/country [Peru]), and there was an average of 23,672 subjects/country (range, 7813 [Côte d’Ivoire, 3 surveys] to 69,300 subjects/country [Peru, 7 surveys]) (Appendix Table 1).

### 2.2 Outcomes

The outcome variable was HAZ of children aged 0–59 months. The z-scores were part of the original data sets and based on the World Health Organization (2006) growth standards. Extreme values (HAZ < −6 or > 6) were discarded. For all subjects, average HAZ was −1.56 (range, −2.32 [average in Bangladesh 1997] to −0.47 [average in Egypt 2014]) (Appendix Table 1).

### 2.3 Covariates

For time stability testing, we selected 14 covariates that were identified as relevant for child growth in previous research (Rieger & Trommlerová, 2016). The covariates covered four levels (with the hypothesized sign of correlation indicated with +/−/~): child (female (~), birth order (~)); parents (maternal height (+), age at birth (~), age at first marriage (+), fertility (−), and education (+), as well as paternal education (+)); household (household size (−), wealth (+)); and community and infrastructure (average access to any toilet in the primary sampling unit (+), mortality of under-5 children in the previous five years in the primary sampling unit (−); household access to electricity (+), rural area of residence (−)). There were seven binary variables (female child, firstborn child, no maternal education, no paternal education, household electricity, household in the lowest wealth quintile, rural household), and the remaining seven variables were continuous. There were two variables with missing values for never-married women (age at first marriage, husband’s education); these missing values were replaced by 0 and a binary variable to indicate the addition of this imputation was included, which kept the coefficients of both variables correctly estimated (the same coefficients as without imputation) and avoided loss of observations.^3^ This was important because systematically excluding never-married women could have caused sample selection bias and made our results less representative of the general population.

We included variables capturing growth faltering (the HAZ-age profile). HAZ in low and middle income countries typically decreases sharply from birth to age 3 years and then stabilizes. This nonlinear pattern was modeled in a segmented piecewise linear regression of HAZ on age, and the slope of age was allowed to differ before and after the cutoffs that varied by geographic region (Latin America, age 20 months; Sub-Saharan Africa and East Asia, 21 months; Middle East and Northern Africa, 22 months; South Asia, 23 months; Central Asia, 28 months). We defined the cutoff as the age with the lowest population-weighted average HAZ, and used region- instead of country-specific cutoffs due to small sample size in some countries. Finally, calendar month of birth was included in the regressions as a set of binary variables to adjust for seasonality. Descriptive statistics were calculated using population weights; models were estimated without any weighting.

### 2.4 Statistical analysis

We tested for the time stability of associations between HAZ and its determinants in two steps using flexible and parsimonious models.

First, the flexible model estimated a separate linear regression for each country. Random effects were set at the primary sampling unit. We allowed the marginal effects of all covariates to vary by survey round by including interactions of each covariate with binary survey round indicators. Thus, we did not impose any constraint on how the marginal effects should change over time. Binary survey round indicators implicitly controlled for all survey-invariant variables such as survey year and years elapsed between two surveys. We tested for the equality of marginal effects associated with a given covariate in the first and last survey round using a Wald test. The null hypothesis was that the marginal effects were statistically equal to each other. We obtained the frequencies at which null hypotheses were rejected by covariate and country. We tested for equality of coefficients in the first versus last survey round (not all rounds) to ensure that all countries were treated identically. For example, if we had tested all rounds, Peru would pass the test only if its coefficients in all 7 survey rounds were statistically equal, in contrast with Chad that has only 2 survey rounds.

Second, the parsimonious model was used to nuance the findings from the flexible model. When a covariate exhibited temporal instability in the flexible model, we proceeded with the parsimonious model and investigated the direction of this instability. We interacted each determinant with a linear time trend to establish whether the marginal effect was increasing or decreasing linearly over survey rounds within a given country. We noted the sign (positive or negative) of the time trend. The parsimonious model was more restrictive than the flexible model because it imposed linear changes in marginal effects over time. The purpose of the parsimonious model was to investigate the underlying deterministic trend direction when covariate associations were unstable in the flexible model.

Standard errors were clustered at the primary sampling unit in all models. Statistical significance was defined by *P* < 0.05.

## 3 Results

Results stem from an analysis of 91 DHS in 25 countries from 1991 to 2014. The outcome variable was height-for-age z-score of children aged 0–59 months, based on WHO growth standards. We estimated two types of random effect models for each country. In the first “flexible” model, we allowed the coefficients of all determinants to vary by survey round. We tested for the equality of these associations between the first and last survey round. In the second “parsimonious” model, we further evaluated the trend of unstable associations across all survey rounds by interacting the covariates with a linear time trend.

### 3.1 Flexible model: temporal stability

**Fig. 1:**
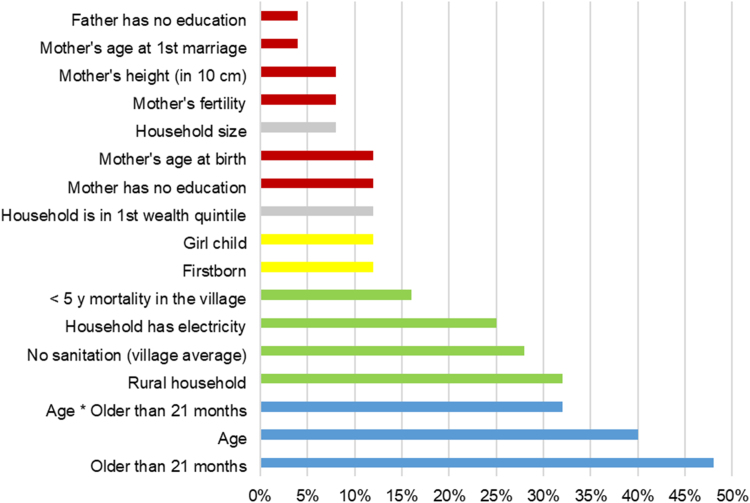
Temporal stability of covariates in Demographic and Health Surveys in 25 countries. *Legend:* Data reported as % countries in which the coefficient associated with the covariate was significantly unstable between the first and last survey as indicated by Wald test (5% significance level). Variable groups: parental (red), household (grey), and child (yellow) characteristics had stable associations with HAZ over time, but community and infrastructure variables (green) were more unstable; variables modeling cross-sectional growth faltering (blue) were the most unstable.

The covariates with the most stable associations with HAZ between the first and last survey were father having no education, mother’s age at first marriage, mother’s height, fertility, and household size; for these variables, associations significantly differed between the first and last survey in at most two out of 25 countries (8%) (Fig. 1). We expected to reject the null hypothesis in 1.25 cases by chance (0.05 significance level × 25 tests); the null hypothesis that the association between father’s education (or mother’s age at first marriage) and HAZ was temporally stable was rejected in only one country (4%). In contrast, we observed one markedly unstable group of indicators that were incorporated in regression analyses to model cross-sectional growth faltering (Rieger & Trommlerová, 2016). In particular, age and age larger 21 months were unstable between survey rounds in 10 (40%) and 12 countries (48%) (Fig. 1), respectively, meaning that the shape of the HAZ-age curve changed from the first to last survey, holding everything else equal. The region-specific cutoff dummy (age larger 21 months) was the most unstable variable (48% countries), confirming national changes in HAZ (upward or downward shifts) in older children. Apart from indicators modelling growth faltering, household residency, access to sanitation, and access to electricity were the most unstable variables (in eight, seven, and six countries, respectively). Classification of variables into individual, household, and community level variables showed that child, parental, and household characteristics had stable associations with HAZ over time, but community and infrastructure variables were more unstable (Fig. 1).

**Fig. 2:**
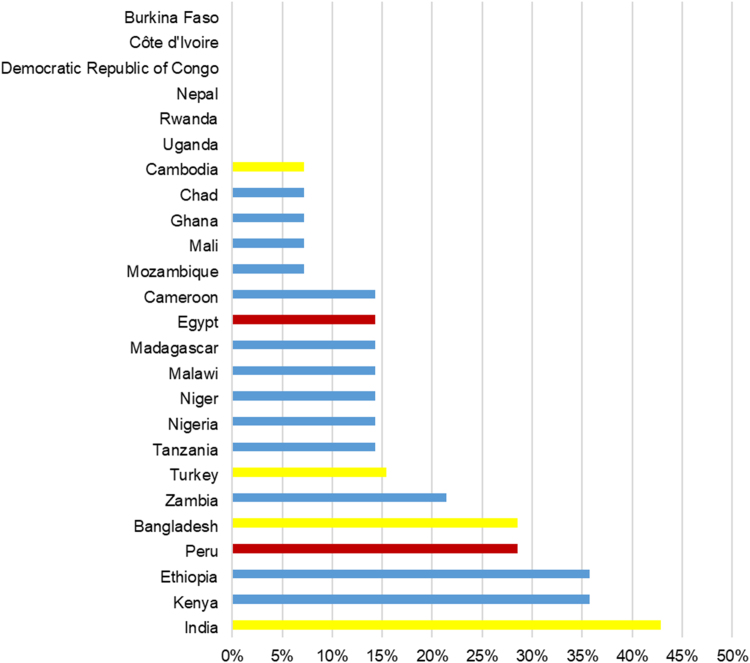
Ranking of 25 countries by the temporal stability of coefficients in Demographic and Health Surveys. *Legend:* Data reported as % covariates with significantly unstable coefficients between the first and last survey in each country as indicated by Wald test (5% significance level), excluding growth faltering variables. Country groups: yellow, Asia; blue, Sub-Saharan Africa; red, other. There was no apparent regional grouping or geographic pattern of temporal stability.

The determinants of HAZ were stable in Burkina Faso, Côte d’Ivoire, Democratic Republic of Congo, Nepal, Rwanda, and Uganda, where none of the 14 coefficients varied in their size at 5% level of significance (Fig. 2). In 19 of the 25 countries (76%), at most two of the 14 covariates were unstable. In contrast, India, Ethiopia, and Kenya had unstable associations between HAZ and the covariates, where six, five, and five of the 14 covariates (43%, 36%, 36%) were unstable, respectively. No single country had instability of over 50% covariates. In the following, we refer to low (high) fraction of time stable coefficient in a country as time stability (instability).

There was no correlation between time instability of covariates and time instability of growth faltering (correlation coefficient 0.21; *P* = 0.32) (Appendix Fig. 1). There was no apparent regional grouping or geographic pattern of temporal stability (Fig. 2).

**Fig. 3:**
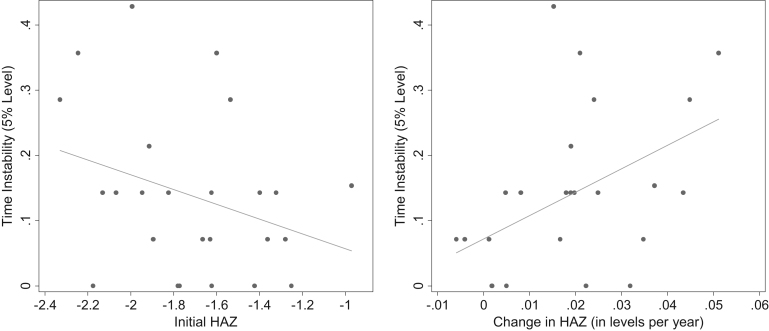
Time instability of the flexible model versus initial and change in height-for-age z-score in Demographic and Health Surveys in 25 countries. *Legend:* Each country represents a point in each graph. Y-axes: % covariates with significantly unstable coefficients between the first and last survey in each country (5% significance level), excluding growth faltering variables. X-axes: left, HAZ in the first survey; right, average annual change in HAZ between first and last survey. Abbreviation: HAZ, height-for-age z-score.

For the 25 countries, the flexible model indicated that temporal instability in the country was negatively but insignificantly correlated with HAZ in the initial survey round (correlation coefficient −0.32; *P* = 0.12) (Fig. 3). In contrast, temporal instability in the country was positively correlated with average annual change in HAZ in the country (correlation coefficient 0.46; *P* = 0.02). Countries that had larger HAZ improvements over time typically had a greater number of unstable covariates. Ethiopia had the highest improvement of all countries in HAZ (0.05 annually) between 2000 and 2011, and had 36% determinants with unstable coefficients. Chad had no change in HAZ between 1996 and 2004 (0.00 annually) and only 7% unstable coefficients. Similarly, the only two countries with negative (and low) change in HAZ, Mali and Mozambique (−0.01 and −0.00 annually, respectively), had 7% of unstable coefficients. The full results of the flexible model are presented in Appendix Table 2.

### 3.2 Flexible and parsimonious models – temporal trend analysis

When the flexible model suggested temporal instability of the associations between covariates and HAZ (5% significance level), we used the parsimonious model to evaluate underlying trends. In what follows and for the sake of brevity, we focus on the covariates with the most stable and least stable coefficients (paternal education and rural residency, respectively), and selected covariates of interest (maternal height and education, access to electricity). Tables with all remaining covariates can be found in the Appendix (Appendix Table 3–11).

First, paternal education was one of the two most temporally stable coefficients, showing temporal instability in one of 25 countries (Fig. 1). All significant coefficients of father having no education were negative, with the exception of one counterintuitively positive coefficient in Kenya (Table 1). In Tanzania, where the coefficient was unstable over time, the parsimonious model showed that the coefficient of no paternal education became less negative over time until it became statistically indistinguishable from zero; it increased from −0.14 (*P* < .01) in 1996 to −0.10 (*P* < .01) in 2004 to 0.02 (insignificant) in 2010.

**Table 1:** Results of flexible and parsimonious models for paternal education in Demographic and Health Surveys in 25 countries.

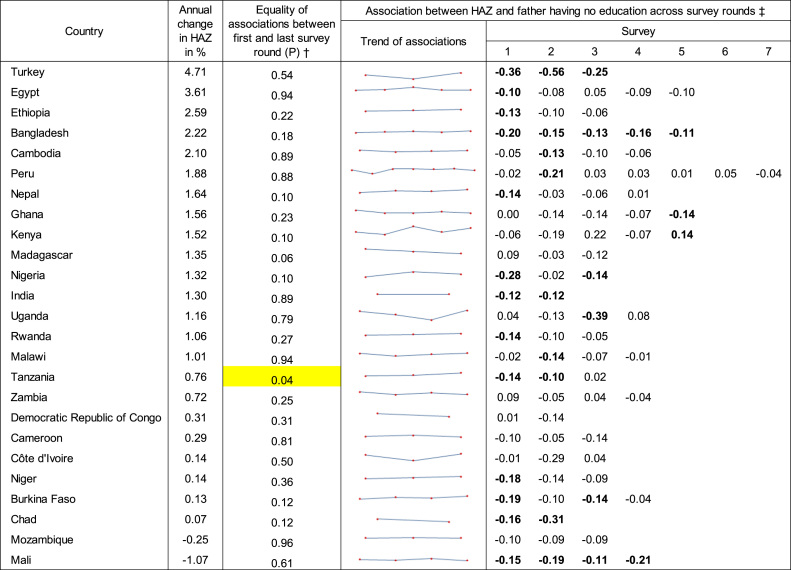

*Legend:* Countries are ranked by annual percentage changes in HAZ between the first and last survey. †P associated with temporal stability test of paternal education between the first and last survey from the flexible model. For Tanzania (P < .05 in the flexible model), the parsimonious model showed linearly increasing (yellow) association of lack of paternal education with HAZ over survey rounds. ‡Estimated associations by survey round from flexible model. Coefficients significant (5% level) are indicated in bold. Abbreviations: HAZ, height-for-age z-score.

In contrast, rural residency had the highest temporal instability of all covariates apart from the HAZ-age profile indicators (Fig. 1), and was temporally unstable (5% significance level) in eight of 25 countries (Table 2). All significant coefficients were negative, and the parsimonious model showed that the coefficient of rural residence became more negative over time in three countries and less negative over time in five countries (Table 2). Ethiopia, Mali, and Peru had increasing strength of the negative association over time; Egypt, India, Malawi, Mozambique, and Zambia had a decrease in the negative association over time (Table 2). For instance, in Mozambique, the rural-urban divide was disappearing since the late 1990s; the negative association between rural residence and HAZ became weaker, increasing from −0.42 (*P* < 0.01) in 1997 to −0.28 (*P* < 0.01) in 2003 and −0.06 (insignificant) in 2011.

**Table 2:** Results of flexible and parsimonious models for rural residency in Demographic and Health Surveys in 25 countries.

*Legend:* Countries are ranked by annual percentage changes in HAZ between the first and last survey. †P associated with temporal stability test of rural residency between the first and last survey for the flexible model. For countries that had P < .05 in the flexible model, the parsimonious model showed linearly increasing (yellow) or decreasing (blue) association of rural residency with HAZ over survey rounds. ‡Estimated associations by survey round from flexible model. Coefficients significant (5% level) are indicated in bold. Abbreviations: HAZ, height-for-age z-score.

In terms of other covariates of interest, maternal height was significantly associated with HAZ in all 91 surveys and was highly stable (Fig. 1). The null hypothesis of equality of coefficients between the first and last survey was rejected only for Madagascar and Zambia (flexible model). There, the coefficient size linearly decreased over time (parsimonious model). In particular, the coefficient of maternal height (measured in 10 cm units) in Madagascar decreased between the first and the last survey round from 0.55 HAZ (year 1997) to 0.41 HAZ (year 2008), and similarly in Zambia from 0.53 HAZ (year 1996) to 0.43 HAZ (year 2013) (Table 3). Overall, the coefficients of maternal height were positive, comparable in size, and highly significant in all countries and surveys (from 0.29 in Egypt to 0.65 in Bangladesh) (Table 3).

**Table 3:** Results of flexible and parsimonious models for maternal height in Demographic and Health Surveys in 25 countries.

*Legend:* Countries are ranked by annual percentage changes in HAZ between the first and last survey. †P associated with temporal stability test of maternal height between the first and last survey from the flexible model. For Madagascar and Zambia (P < .05 in the flexible model), the parsimonious model showed linearly decreasing (blue) association of maternal height with HAZ over survey rounds. ‡Estimated associations by survey round from flexible model. Coefficients significant (5% level) are indicated in bold. All coefficients were significant. Abbreviations: HAZ, height-for-age z-score.

Maternal education, which is an important determinant of child health, was the most unstable *parental* covariate, together with maternal age at birth (Fig. 1). Similarly to paternal education, all significant coefficients of mother having no education were negative, again with the exception of one counterintuitively positive coefficient in Kenya (Table 4). The coefficient was temporally unstable in three of 25 countries: Cambodia, India, and Kenya. There, the parsimonious model indicated a linear increase in this coefficient over time. However, the meaning of this increase differed. In India, the coefficient became less negative but remained significant (increase from -0.35 in year 1998 to -0.21 in year 2005) while in Cambodia, the coefficient became less negative until indistinguishable from zero (increase from -0.17 in year 2000 to insignificant 0.12 in year 2014). Conversely, in Kenya, the coefficient increased from insignificant -0.05 in 1993 to the counterintuitive positive coefficient of 0.19 in 2014.

**Table 4:** Results of flexible and parsimonious models for maternal education in Demographic and Health Surveys in 25 countries.

*Legend:* Countries are ranked by annual percentage changes in HAZ between the first and last survey. †P associated with temporal stability test of maternal education between the first and last survey from the flexible model. For Cambodia, Kenya, and India (P < .05 in the flexible model), the parsimonious model showed linearly increasing (yellow) association of lack of maternal education with HAZ over survey rounds. ‡Estimated associations by survey round from flexible model. Coefficients significant (5% level) are indicated in bold. Abbreviations: HAZ, height-for-age z-score.

Finally, electricity as an infrastructure covariate was temporally unstable in six of 25 countries. If significant, it was always positively associated with HAZ, reaching from 0.10 in India to 0.97 in Rwanda (Table 5). In all six countries where it was temporally unstable in the flexible model, the parsimonious model showed that the magnitude of the coefficient was decreasing over time. In three countries (Bangladesh, Ethiopia, Kenya), the magnitude of the coefficient decreased over time but remained positive while in the remaining three countries (Malawi, Niger, Peru), the coefficient became insignificant in the last survey round.

**Table 5:** Results of flexible and parsimonious models for electricity in Demographic and Health Surveys in 25 countries.

*Legend:* Countries are ranked by annual percentage changes in HAZ between the first and last survey. †P associated with temporal stability test of electricity between the first and last survey from the flexible model. For Ethiopia, Bangladesh, Peru, Kenya, Malawi, and Niger (P < .05 in the flexible model), the parsimonious model showed linearly decreasing (blue) association of electricity with HAZ over survey rounds. ‡Estimated associations by survey round from flexible model. Coefficients significant (5% level) are indicated in bold. DHS in Turkey did not collect information on electricity. Abbreviations: HAZ, height-for-age z-score.

### 3.3 Further sensitivity analysis

In the main analysis, we used all available surveys in the estimations but tested for temporal stability of coefficients using only the first and last survey round coefficients, thus assuring consistent treatment of all countries independently of the number of survey rounds available. Had we tested time stability using all survey rounds coefficients, availability of more survey rounds in a country would have led to more coefficients for comparison, which may have increased the probability that coefficients would be statistically unequal over survey rounds in this country. However, when testing temporal stability over all survey rounds in a robustness check, countries with many surveys did not necessarily become more unstable; while we observed an increase in instability in Egypt (five rounds) and Peru (seven rounds) when all rounds were considered, there was no change in instability in Bangladesh (five rounds), and Kenya and Ghana (five rounds each) showed decreased instability (Appendix Fig. 2).

There was a high correlation between the number of survey rounds and time between the first and last survey (correlation coefficient 0.84; *P* < 0.01) (Appendix Fig. 3). Nevertheless, the time between the first and last survey in a country did not affect the time stability of covariates (correlation coefficient 0.02; *P* = 0.94).

### 3.4 Extensions

Fig. 1 documented that growth faltering indicators were unstable over time in 40–48% of countries, meaning that the shape of the HAZ-age curve changed significantly over time in almost half of the countries. This is consistent with a hypothesis of a secular change (increase or decrease) in HAZ at a given age over time. We found supporting evidence for this hypothesis in the parsimonious model, where the variables were interacted with a linear time trend. We found that coefficients of both age and age larger 21 months were significantly increasing over time in 10 countries each (40%), meaning that there were secular improvements in HAZ over time (Table 6 and Appendix Table 12). In three countries, either coefficient of age (Chad) or coefficient of age larger 21 months (Ethiopia, India) linearly *decreased* over time.

**Table 6:** Results of flexible and parsimonious models for age in the first 21 months in Demographic and Health Surveys in 25 countries.

*Legend:* Countries are ranked by annual percentage changes in HAZ between the first and last survey. †P associated with temporal stability test of age in the first 21 months between the first and last survey from the flexible model. For countries that had P < .05 in the flexible model, the parsimonious model showed linearly increasing (yellow) or decreasing (blue) association of age in the first 21 months with HAZ over survey rounds. In addition, Nigeria showed a linearly increasing association in the parsimonious model, despite having P > .05 in the flexible model. ‡Estimated associations by survey round from flexible model. Coefficients significant (5% level) are indicated in bold. Abbreviations: HAZ, height-for-age z-score.

In another extension, we explored the spatial stability of coefficients. We estimated a flexible model which included all countries in one linear regression. As before, random effects were set at the primary sampling unit. We added country-year binary indicators which controlled for all survey-invariant variables, such as survey year, and all time-invariant country characteristics. We allowed the marginal effects of all covariates to vary by country through inclusion of interaction terms between each covariate and binary country indicators. We tested for the equality of marginal effects associated with a given covariate across all 25 countries using a Wald test. The null hypothesis was that the marginal effects were statistically equal to each other. We found that all covariates except for one (mortality of under-5 children) were statistically different in the 25 countries. This could have been driven by the large geographical coverage in our sample and by the large number of coefficients tested (25 for each covariate). We then restricted the model to one region only (Sub-Saharan Africa, 18 countries) and we found the same result: coefficients of 13 out of 14 covariates varied between the countries significantly; only coefficient of mortality of under-5 children did not. Also this result could have been driven by the large number of coefficients tested (18 for each covariate). We then estimated a simplified version of this model where we interacted each covariate with two regional binary indicators (instead of 25 country binary indicators): Sub-Saharan Africa (includes 18 countries), and “other regions” (7 countries). In this set-up, 9 coefficients differed statistically between Sub-Saharan Africa and other regions, while 5 coefficients were statistically indistinguishable (father has no education, mother’s age at first marriage, household size, household wealth, and mortality of under-5 children).

Finally, HAZ is a measure of long-term child growth while weight-for-height z-score (WHZ) captures short-term changes in the nutritional and health status of a child. We examined temporal stability of associations between the same set of covariates and WHZ. We estimated the same models as for HAZ with only one alteration: the relationship between WHZ and age was modeled as cubic since there is no growth faltering pattern in WHZ. The results indicated that among the chosen set of covariates, there was less temporal instability of associations between the covariates and WHZ than was the case for HAZ (Appendix Fig. 4). There was no clear grouping of covariates in terms of their temporal stability. The most unstable covariates were access to sanitation (48% of countries), household wealth (20%), and sex of the child (20%). The most stable covariates were access to electricity and birth order (both 0%).

## 4 Discussion

The present evaluation of the temporal evolution of associations between demographic and socio-economic variables and child growth used a two-pronged testing procedure (testing for the equality of associations between two survey rounds, and estimating the linear trend of unstable associations). Using nationally representative data from multiple countries with different ranges between the survey years (6–21 years), the evaluation was able to apply the same time stability tests in diverse geographic areas.

The overall associations between HAZ and common determinants of child growth were constant over the years tested; excluding growth faltering covariates, 12 out of 14 covariates were stable in 19 of 25 countries (76%) (Fig. 2). This finding is consistent with recent studies with similar temporal stability tests for select Asian countries (Headey et al., 2015, Headey et al., 2016, Rabbani et al., 2016, Headey and Hoddinott, 2015). When associations were unstable, we classified instability in positively or negatively trending cases. We observed that the rural-urban gap was increasing in some and decreasing in other countries (Table 2), consistent with other studies about the role of household residency for child health (Hathi et al., 2017, Fotso, 2007, Van de Poel et al., 2007). One partial explanation for these patterns is that rural residence is measured with less consistency than most other measures in the DHS data. Since countries classify urban versus rural areas, definitions may vary across countries and may change over time within countries. We also identified the most stable determinants of HAZ, which were paternal education, maternal age at marriage, height, and fertility, and household size (Fig. 1). The most unstable variables were indicators modelling growth faltering, followed by household residency, access to sanitation, and access to electricity.

In general, we found that child growth displayed relatively more time stable associations with child, parental, and household factors than with community and infrastructure factors. This might be related to the fact that community and infrastructure factors themselves might change more over time than child, parental, and household factors. We tested this hypothesis by looking at the correlation between time stability of a covariate and its change over time. We included 11 of 14 covariates where changes over time are expected (for child’s sex, birth order, and household wealth quintile we expect no changes over time). As a measure of temporal stability, we calculated the average p-value of each covariate in Wald test across 25 countries; higher average p-value means more temporal stability. As a measure of changes over time, we calculated the average absolute change in the covariate between the first and last survey round; we calculated both a change in percent and a change in percent per year to account for different time spans in the different countries. The correlation coefficient between the average p-value and the average absolute change in the covariate in percent was negative and statistically insignificant (correlation coefficient −0.39; *P* = 0.24). When considering change in the covariate in percent per year, the results did not change (correlation coefficient −0.34; *P* = 0.31). The magnitude of the correlation is substantial but mainly driven by electricity which is an outlier in terms of changes over time, and is temporally unstable. When excluding electricity, the correlation coefficient reduces substantially and remains statistically insignificant (correlation coefficient for change in covariate in percent −0.09; *P* = 0.80; correlation coefficient for change in covariate in percent per year −0.17; *P* = 0.63).

We observed moderate heterogeneity in coefficient stability between countries. India, Ethiopia, and Kenya had the highest number of unstable coefficient sizes over time, but instability was limited even in these countries (maximum 43% of coefficients) (Fig. 2). Further study is justified for countries that displayed elevated temporal instability.

In countries with many survey rounds, both increased and decreased instability was observed when equality across *all* surveys was tested (Appendix Fig. 2). Countries with increasing temporal instability when *all* surveys were tested may have had coefficients changing in both directions over time (first increasing, then decreasing), and the potential to detect instability may have increased with more survey rounds. In contrast, countries with decreasing temporal instability when coefficients from *all* survey rounds were tested may have had a gradual change in the coefficients between the survey rounds, and the Wald test may have evaluated the coefficients as similar among all surveys, even though the change in the coefficients between the first and last round may have been substantial and significant. Overall, the ranking of countries would have changed if all coefficients were tested simultaneously; this justifies the treatment of all countries equally by testing the coefficients from only two rounds per country. However, this approach could be problematic as countries with more available surveys exhibit a larger time gap between the first and last survey which could in theory give more scope for demonstrating a temporal instability. As we do not find any systematic relationship between temporal instability and time between the first and last survey, our approach is corroborated (Appendix Fig. 3).

Although we tested only two survey rounds per country in our main specification, the flexible model was estimated using all survey rounds. All other factors unchanged, this caused larger sample sizes in countries with more surveys, which may have increased the detectability of temporal instabilities there. We re-estimated the flexible models using only the first and last survey rounds to verify sensitivity; the results regarding temporal instability of both covariates and countries were extremely similar (results not shown).

Our study is the first to conduct a cross-country examination of temporal model stability. To compare, another study (Headey et al., 2015) with several rounds of DHS data from Bangladesh, different model specification, and different testing approach showed similarly stable coefficient sizes (4 of 13 covariates were unstable) as were observed in Bangladesh in the present study (4 of 14 coefficients were unstable).

We also observed that indicators used to model HAZ-age profile (cross-sectional growth faltering) were the most unstable covariates. This is consistent with the previous DHS study in Bangladesh (Headey et al., 2015). At the same time, important improvements in HAZ (27%) occurred in Bangladesh between 1997 and 2011 (Appendix Table 1). This suggests that secular changes in HAZ might have taken place at a given age over time: as child health improved, the shape of the HAZ-age profile changed. We found supporting evidence for the hypothesis of secular improvements in HAZ over time; coefficients of both age and age larger 21 months were significantly increasing over time in 40% of countries in the parsimonious model (Table 6 and Appendix Table 12). However, in three countries, either coefficient of age (Chad) or coefficient of age larger 21 months (Ethiopia, India) linearly *decreased* over time. Results in Chad are not surprising as Chad ranked 23 out of 25 countries in terms of average annual changes in HAZ over time. Results in India and especially in Ethiopia are less intuitive because they indicate that there were negative national changes in HAZ among older children despite the fact that these countries ranked 16–17 and 1–8 out of 25 in three different measures of average annual improvements in HAZ, respectively. One possible explanation is that in these countries, inequality in terms of health and nutrition among children of different ages increased.

In addition to temporal stability, we explored also spatial stability of coefficients. When a large number of coefficients was tested in a between-country-stability Wald test (one coefficient for each country, i.e. 25 coefficients per covariate), 13 of 14 covariates were spatially unstable. However, when the number of tested coefficients decreased to two in a between-region-stability test (Sub-Saharan Africa versus other regions), 9 coefficients were unstable between the regions while 5 coefficients were stable. When we contrast temporal and spatial stability of coefficients, we find that the five covariates that had spatially stable coefficients between Sub-Saharan Africa and other regions include two covariates with the most temporally stable coefficients (father has no education, mother’s age at first marriage), both household-level covariates (household size, household wealth), and the most temporally stable community level covariate (mortality of under-5 children).

In addition to temporal stability between child growth determinants and a measure of long-term child growth (HAZ), we explored also their stability with respect to a measure of short-term changes in the nutritional and health status of children (WHZ). We found less temporal instability of coefficients. This might be driven by the fact that we chose covariates which were known determinants of children’s *long*-term growth (HAZ). In the WHZ regressions, their coefficients were significant less often than in HAZ regressions. Hence, lack of temporal instability in WHZ regressions might be a result of covariates not being relevant for WHZ (and their coefficients being insignificant) rather than of the coefficients being stable over time. We found that there was no clear grouping of covariates in terms of their temporal instability, and the most unstable covariates were access to sanitation (48% of countries), household wealth (20%), and sex of the child (20%). Access to sanitation is the most relevant covariate in WHZ regressions as it has most significant coefficients of the 14 covariates. This supports the hypothesis that the temporal instability in WHZ regressions might reflect the covariates being irrelevant rather than their coefficients being unstable. Another interesting finding is that access to electricity, which was the third most unstable covariate in HAZ regressions, displayed no temporal instability in WHZ regressions.

Limitations of the present study included the evaluation of a select set of covariates; we aimed to have comparable indicators between countries, rather than an exhaustive list of covariates, but other omitted factors may have important temporal instability. In addition, we did not determine the underlying mechanisms when association instability was identified. Furthermore, DHS surveys were not implemented at strictly regular intervals, and some countries in our sample had more rounds that were performed over a longer period than others. We addressed these data issues by testing for temporal instability similarly in all countries.

## 5 Conclusions

The DHS datasets are widely used to estimate the determinants of child growth, which is not surprising given their comparability across countries and time, their quality, scope and coverage. While there have been numerous studies comparing effects across geographies, work on temporal effect stability at the country level was still awaited. The present study provides improved understanding of temporal stability of HAZ models and of regional differences therein. First, we find that associations between HAZ and child, parental, and household characteristics (e.g. paternal education, maternal age at marriage, height, and fertility, and household size) do not change over time whereas coefficients of community and infrastructure variables (e.g. household residency, access to sanitation, and access to electricity) are more unstable. Second, among the unstable coefficients, there is no uniform geographical pattern in terms of consistently increasing or decreasing associations over time. For instance, association between HAZ and rural residency is negative (or zero) in all countries but it decreases over time in some of them while it increases in others (or stays stable in yet another group). Third, indicators of growth faltering are the most unstable over time, mainly confirming secular changes in HAZ at a given age over time, and national changes in HAZ in children older than 2 years. Fourth, countries with the most unstable coefficients are those that started off with lower average HAZ and those that experienced higher improvements in HAZ over time. This is consistent with a convergence model where countries that are initially worse off catch up more quickly and this is either reflected in or driven by changing associations between HAZ and infrastructure and community covariates. Finally, there was no apparent regional grouping or geographic pattern of temporal stability.

These findings may inform about the generalizability of results stemming from cross-sectional studies that do not consider time variation. For instance, results regarding effects of child, parental, and household factors on HAZ do not necessarily need to be re-evaluated over time whereas results regarding the effects of infrastructure and community variables need to be monitored more frequently as they are expected to change. A priori, it is unclear in which direction the change will go (no uniform pattern was found) and changes observed in one country cannot be assumed to hold in the neighboring country (no regional groupings in terms of trends in associations were found). In addition, the study may further the Precision Public Health approach to solving global stunting by producing knowledge that may improve population targeting of interventions in different regions and times. The present results suggest that temporal differences in community and infrastructure factors, such as access to sanitation and electricity, are important for Precision Public Health population targeting in child growth; for child, parental, and household factors, temporal targeting seems not to be relevant. However, findings from one country should not be assumed as true even for countries within the same region as we found no regional patterns. More in general, regional targeting might be of importance for all factors and warrants further systematic investigation.

## Footnotes

2 In a systematic literature review, no previous global or systematic investigation of the temporal stability of regression coefficients of common socio-economic determinants of child growth and height-for-age z-score was found. In particular, we searched PubMed for articles published in English between January 1, 2000 and January 1, 2017. We searched in titles and abstracts, using the terms “child growth” or “child stunting” or “height-for-age” and “structural break” or “temporal stability” or “effect stability” or “time stability” or “association stability” or “model stability.” The 2 studies identified were not relevant to the present study.

3 This approach yields correctly estimated coefficients of age at marriage and husband’s education independently of the choice of the imputed value, as long as a binary variable indicating that an imputation was made is included in the model.
