# Supplementary material for: Temporal stability of child growth associations in Demographic and Health Surveys in 25 countries
Source: SSM Popul Health. 2019 Jan 12;7:100352. doi: 10.1016/j.ssmph.2019.100352 (PMC6351606; doi:10.1016/j.ssmph.2019.100352)
Supplement: Supplementary file 1 — Supplementary material [file mmc1.pdf]

Appendix

Appendix Table 1. Summary statistics of height-for-age z-score and sample sizes in 91 Demographic and Health Surveys in 25 countries used in the analysis of temporal stability of child growth correlates

Country	Year	HAZ	Sample size		Number of surveys
			Per survey	Per country	
Bangladesh	1997	-2.32	4615	27 415	5
	2000	-2.00	5229		
	2004	-1.95	5902		
	2007	-1.74	4784		
	2011	-1.69	6885		
Burkina Faso	1993	-1.33	3917	21 838	4
	1998	-1.56	3530		
	2003	-1.64	7937		
	2010	-1.39	6454		
Cambodia	2000	-1.95	3479	14 954	4
	2005	-1.86	3547		
	2010	-1.65	3650		
	2014	-1.40	4278		
Cameroon	1998	-1.28	1642	9443	3
	2004	-1.40	2988		
	2011	-1.25	4813		
Chad	1996	-1.61	5453	9716	2
	2004	-1.57	4263		
Democratic Republic of Congo	2007	-1.62	3165	11 047	2
	2013	-1.66	7882		
Côte d'Ivoire	1994	-1.24	3345	7813	3
	1998	-1.21	1378		
	2011	-1.25	3090		
Egypt	1995	-1.38	10 230	53 742	5
	2000	-1.03	9907		
	2005	-1.04	11 647		
	2008	-1.00	8996		
	2014	-0.47	12 962		
Ethiopia	2000	-2.07	8333	21 524	3
	2005	-1.79	3800		
	2011	-1.61	9391		
Ghana	1993	-1.28	1811	12 421	5
	1998	-1.35	2608		

	2003	-1.44	3005		
	2008	-1.08	2344		
	2014	-0.98	2653		
India	1998	-1.91	24 759	63 277	2
	2005	-1.72	38 518		
Kenya	1993	-1.57	4738	25 901	5
	1998	-1.40	2886		
	2003	-1.34	4491		
	2008	-1.35	4981		
	2014	-1.18	8805		
Madagascar	1997	-2.02	2875	11 988	3
	2003	-1.90	4317		
	2008	-1.78	4796		
Malawi	1992	-2.02	3148	24 454	4
	2000	-1.98	8891		
	2004	-1.97	7930		
	2010	-1.77	4485		
Mali	1995	-1.38	4597	28 528	4
	2001	-1.63	9046		
	2006	-1.43	10 636		
	2012	-1.43	4249		
Mozambique	1997	-1.65	3055	20 023	3
	2003	-1.85	7780		
	2011	-1.58	9188		
Nepal	1996	-2.17	3708	16 744	4
	2001	-2.20	5868		
	2006	-1.98	4960		
	2011	-1.74	2208		
Niger	1998	-1.77	3839	12 169	3
	2006	-1.95	3642		
	2012	-1.68	4688		
Nigeria	2003	-1.60	4184	46 996	3
	2008	-1.55	18 645		
	2013	-1.34	24 167		
Peru	1991	-1.59	7451	69 300	7
	1996	-1.46	14 687		
	2000	-1.47	11 185		
	2006	-1.36	10 117		
	2010	-1.28	8457		
	2011	-1.22	8476		
	2012	-1.15	8927		
Rwanda	2000	-1.72	5964	13 612	3
	2005	-1.90	3614		

	2010	-1.75	4034		
Tanzania	1996<br>2004<br>2010	-1.96<br>-1.75<br>-1.64	5106<br>6857<br>6530	18 493	3
Turkey	1993<br>1998<br>2003	-0.94<br>-0.84<br>-0.69	3121<br>2769<br>4017	9907	3
Uganda	1995<br>2001<br>2006<br>2011	-1.67<br>-1.76<br>-1.53<br>-1.39	4462<br>4898<br>2275<br>2026	13 661	4
Zambia	1996<br>2001<br>2007<br>2013	-1.96<br>-2.06<br>-1.65<br>-1.58	5471<br>5249<br>5000<br>11 116	26 836	4

Appendix Table 3. Results of flexible and parsimonious models for maternal age at marriage in Demographic and Health Surveys in 25 countries

Country	Annual change in HAZ in %	Equality of associations between first and last survey round (P) †	Association between HAZ and mother's age at first marriage across survey rounds ‡							
			Trend of associations	Survey						
				1	2	3	4	5	6	7
Turkey	4.71	0.31		**-0.03**	-0.02	-0.02				
Egypt	3.61	0.80		0.00	0.00	**-0.01**	**-0.02**	0.00		
Ethiopia	2.59	0.42		0.01	0.00	0.00				
Bangladesh	2.22	0.08		0.00	0.01	0.00	-0.01	**0.02**		
Cambodia	2.10	0.06		**-0.03**	**-0.02**	-0.01	-0.01			
Peru	1.88	0.12		**-0.01**	**-0.01**	0.00	**-0.01**	**-0.01**	**-0.01**	0.00
Nepal	1.64	0.35		0.00	-0.01	0.00	-0.01			
Ghana	1.56	0.08		**-0.03**	-0.01	**-0.03**	-0.01	0.00		
Kenya	1.52	0.23		0.00	0.00	0.00	0.00	**-0.02**		
Madagascar	1.35	0.87		0.00	0.00	0.00				
Nigeria	1.32	0.27		**0.02**	0.00	**0.01**				
India	1.30	0.01		**0.02**	0.00					
Uganda	1.16	0.50		0.01	0.00	-0.02	0.00			
Rwanda	1.06	0.05		0.01	**-0.02**	-0.01				
Malawi	1.01	0.06		-0.01	-0.01	**-0.03**	**-0.03**			
Tanzania	0.76	0.77		**-0.02**	**-0.01**	**-0.01**				
Zambia	0.72	0.86		0.00	-0.02	-0.01	0.00			
Democratic Republic of Congo	0.31	0.16		0.00	**-0.02**					
Cameroon	0.29	0.28		-0.01	-0.01	0.01				
Côte d'Ivoire	0.14	0.25		0.00	0.00	**-0.02**				
Niger	0.14	0.93		-0.01	0.02	-0.01				
Burkina Faso	0.13	0.78		-0.01	0.02	-0.01	-0.02			
Chad	0.07	0.69		0.02	0.01					
Mozambique	-0.25	0.68		0.00	-0.01	0.00				
Mali	-1.07	0.77		-0.01	0.00	0.00	-0.02			

Legend: Countries are ranked by annual percentage changes in HAZ between the first and last survey. †P associated with temporal stability test of maternal age at marriage between the first and last survey from the flexible model. For India (P < .05 in the flexible model), the parsimonious model showed linearly decreasing (blue) association of maternal age at marriage with HAZ over survey rounds. ‡Estimated associations by survey round from flexible model. Coefficients significant (5% level) are indicated in bold. Abbreviations: HAZ, height-for-age z-score.

Appendix Table 4. Results of flexible and parsimonious models for fertility in Demographic and Health Surveys in 25 countries

Country	Annual change in HAZ in %	Equality of associations between first and last survey round (P) †	Association between HAZ and mother's fertility across survey rounds ‡							
			Trend of associations	Survey						
				1	2	3	4	5	6	7
Turkey	4.71	0.02		**-0.12**	**-0.09**	**-0.05**				
Egypt	3.61	0.60		-0.01	-0.03	**-0.05**	-0.04	-0.03		
Ethiopia	2.59	0.03		-0.03	0.02	0.02				
Bangladesh	2.22	0.76		**-0.09**	**-0.12**	**-0.14**	**-0.12**	**-0.10**		
Cambodia	2.10	0.35		**-0.05**	**-0.07**	**-0.06**	**-0.08**			
Peru	1.88	0.08		**-0.11**	**-0.11**	**-0.09**	**-0.12**	**-0.12**	**-0.09**	**-0.09**
Nepal	1.64	0.14		**-0.04**	**-0.06**	**-0.06**	**-0.09**			
Ghana	1.56	0.84		**-0.09**	**-0.09**	**-0.09**	-0.03	**-0.10**		
Kenya	1.52	0.32		**-0.05**	-0.02	**-0.06**	0.00	**-0.08**		
Madagascar	1.35	0.67		**-0.05**	**-0.04**	**-0.06**				
Nigeria	1.32	0.10		0.00	-0.01	**-0.03**				
India	1.30	0.47		**-0.07**	**-0.08**					
Uganda	1.16	0.14		-0.03	**-0.06**	**-0.06**	**-0.09**			
Rwanda	1.06	0.13		**-0.05**	**-0.08**	**-0.10**				
Malawi	1.01	0.10		-0.02	**-0.03**	**-0.07**	**-0.08**			
Tanzania	0.76	0.65		**-0.04**	**-0.04**	-0.03				
Zambia	0.72	0.27		-0.02	**-0.08**	**-0.05**	**-0.05**			
Democratic Republic of Congo	0.31	0.15		-0.04	**-0.08**					
Cameroon	0.29	0.27		**-0.07**	**-0.06**	-0.03				
Côte d'Ivoire	0.14	0.20		-0.02	-0.03	**-0.05**				
Niger	0.14	0.97		**-0.05**	**-0.06**	**-0.05**				
Burkina Faso	0.13	0.82		**-0.06**	**-0.04**	**-0.05**	**-0.06**			
Chad	0.07	0.34		0.00	0.03					
Mozambique	-0.25	0.35		-0.04	**-0.04**	**-0.07**				
Mali	-1.07	0.65		**-0.04**	-0.01	-0.03	-0.03			

Legend: Countries are ranked by annual percentage changes in HAZ between the first and last survey. †P associated with temporal stability test of fertility between the first and last survey from the flexible model. For Turkey and Ethiopia (P < .05 in the flexible model), the parsimonious model showed linearly increasing (yellow) association of fertility with HAZ over survey rounds. ‡Estimated associations by survey round from flexible model. Coefficients significant (5% level) are indicated in bold. Abbreviations: HAZ, height-for-age z-score.

Appendix Table 5. Results of flexible and parsimonious models for household size in Demographic and Health Surveys in 25 countries

Country	Annual change in HAZ in %	Equality of associations between first and last survey round (P) †	Association between HAZ and household size across survey rounds ‡							
			Trend of associations	Survey						
				1	2	3	4	5	6	7
Turkey	4.71	0.73		**-0.03**	0.00	**-0.03**				
Egypt	3.61	0.10		-0.01	0.00	0.00	0.01	0.01		
Ethiopia	2.59	0.80		0.01	-0.03	0.01				
Bangladesh	2.22	0.13		0.01	-0.01	0.00	0.01	0.00		
Cambodia	2.10	0.22		-0.03	0.00	-0.01	0.00			
Peru	1.88	0.06		**-0.02**	**-0.02**	**-0.02**	**-0.02**	**-0.01**	-0.01	-0.01
Nepal	1.64	0.56		0.00	0.00	0.00	0.01			
Ghana	1.56	0.33		0.00	0.00	0.01	-0.01	**-0.02**		
Kenya	1.52	0.94		-0.01	-0.02	0.00	**-0.03**	-0.01		
Madagascar	1.35	0.29		0.01	-0.02	-0.01				
Nigeria	1.32	0.04		**-0.03**	-0.01	**-0.01**				
India	1.30	0.01		**-0.01**	0.00					
Uganda	1.16	0.46		-0.01	0.01	0.01	0.00			
Rwanda	1.06	0.05		**0.07**	0.00	0.03				
Malawi	1.01	0.43		-0.01	0.00	0.01	0.00			
Tanzania	0.76	0.50		-0.01	0.01	0.00				
Zambia	0.72	0.67		0.00	**-0.02**	0.00	0.00			
Democratic Republic of Congo	0.31	0.30		0.01	-0.01					
Cameroon	0.29	0.33		0.00	**-0.02**	0.01				
Côte d'Ivoire	0.14	0.13		0.00	0.00	**-0.02**				
Niger	0.14	0.77		0.00	0.00	-0.01				
Burkina Faso	0.13	0.96		-0.01	0.01	0.00	-0.01			
Chad	0.07	0.28		0.00	-0.01					
Mozambique	-0.25	0.51		0.00	**-0.01**	0.01				
Mali	-1.07	0.30		**-0.02**	**-0.01**	**-0.01**	0.00			

Legend: Countries are ranked by annual percentage changes in HAZ between the first and last survey. †P associated with temporal stability test of household size between the first and last survey from the flexible model. For countries that had P < .05 in the flexible model, the parsimonious model showed linearly increasing association (yellow) or no linear trend in the association (grey) of household size with HAZ over survey rounds. ‡Estimated associations by survey round from flexible model. Coefficients significant (5% level) are indicated in bold. Abbreviations: HAZ, height-for-age z-score.

Appendix Table 6. Results of flexible and parsimonious models for maternal age at birth in Demographic and Health Surveys in 25 countries

Country	Annual change in HAZ in %	Equality of associations between first and last survey round (P) †	Association between HAZ and mother's age at birth across survey rounds ‡							
			Trend of associations	Survey						
				1	2	3	4	5	6	7
Turkey	4.71	0.00	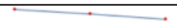	**0.07**	**0.06**	**0.04**				
Egypt	3.61	0.74		0.01	**0.01**	**0.02**	**0.02**	**0.01**		
Ethiopia	2.59	0.04		**0.01**	0.01	0.00				
Bangladesh	2.22	0.78		**0.03**	**0.04**	**0.03**	**0.02**	**0.03**		
Cambodia	2.10	0.16		**0.03**	**0.02**	0.01	**0.02**			
Peru	1.88	0.00		**0.04**	**0.04**	**0.03**	**0.03**	**0.03**	**0.03**	**0.03**
Nepal	1.64	0.36		**0.01**	**0.02**	**0.02**	**0.02**			
Ghana	1.56	0.41		**0.04**	**0.03**	**0.03**	**0.02**	**0.03**		
Kenya	1.52	0.31		**0.04**	**0.03**	**0.03**	0.01	**0.03**		
Madagascar	1.35	0.52		0.01	**0.02**	**0.02**				
Nigeria	1.32	0.70		**0.02**	**0.01**	**0.02**				
India	1.30	0.15		**0.03**	**0.03**					
Uganda	1.16	0.06		**0.02**	**0.03**	**0.02**	**0.05**			
Rwanda	1.06	0.33		0.01	**0.03**	**0.02**				
Malawi	1.01	0.37		0.02	**0.01**	**0.02**	**0.03**			
Tanzania	0.76	0.37		**0.02**	**0.01**	0.01				
Zambia	0.72	0.25		**0.01**	**0.03**	**0.03**	**0.02**			
Democratic Republic of Congo	0.31	0.47		**0.03**	**0.04**					
Cameroon	0.29	0.97		**0.03**	**0.03**	**0.03**				
Côte d'Ivoire	0.14	0.65		**0.02**	**0.03**	**0.02**				
Niger	0.14	0.25		**0.01**	**0.02**	**0.03**				
Burkina Faso	0.13	0.58		**0.03**	**0.02**	**0.02**	**0.03**			
Chad	0.07	0.15		0.01	-0.01					
Mozambique	-0.25	0.34		**0.03**	**0.02**	**0.02**				
Mali	-1.07	0.87		**0.02**	**0.01**	**0.01**	**0.02**			

Legend: Countries are ranked by annual percentage changes in HAZ between the first and last survey. †P associated with temporal stability test of maternal age at birth between the first and last survey from the flexible model. For Turkey, Ethiopia, and Peru (P < .05 in the flexible model), the parsimonious model showed linearly decreasing (blue) association of maternal age at birth with HAZ over survey rounds. ‡Estimated associations by survey round from flexible model. Coefficients significant (5% level) are indicated in bold. Abbreviations: HAZ, height-for-age z-score.

Appendix Table 7. Results of flexible and parsimonious models for household wealth in Demographic and Health Surveys in 25 countries

Country	Annual change in HAZ in %	Equality of associations between first and last survey round (P) †	Association between HAZ and household being in the first wealth quintile across survey rounds ‡							
			Trend of associations	Survey						
				1	2	3	4	5	6	7
Turkey	4.71	0.06		**-0.19**	**-0.22**	**-0.38**				
Egypt	3.61	0.15		0.02	-0.08	**-0.16**	0.00	-0.08		
Ethiopia	2.59	0.06		0.05	-0.07	-0.07				
Bangladesh	2.22	0.00		0.00	**-0.17**	**-0.20**	-0.11	**-0.29**		
Cambodia	2.10	0.47		**-0.15**	-0.09	**-0.19**	-0.09			
Peru	1.88	0.17		**-0.22**	**-0.15**	**-0.19**	**-0.19**	**-0.31**	**-0.31**	**-0.30**
Nepal	1.64	0.31		**-0.11**	**-0.24**	**-0.12**	**-0.21**			
Ghana	1.56	0.02		**-0.25**	-0.12	-0.09	-0.08	0.03		
Kenya	1.52	0.02		**-0.13**	**-0.27**	**-0.21**	**-0.19**	**-0.32**		
Madagascar	1.35	0.84		-0.07	-0.13	-0.09				
Nigeria	1.32	0.07		0.00	**-0.19**	**-0.19**				
India	1.30	0.42		-0.04	**-0.08**					
Uganda	1.16	0.73		-0.12	-0.11	-0.17	-0.16			
Rwanda	1.06	0.17		**-0.13**	**-0.22**	**-0.23**				
Malawi	1.01	0.94		**-0.21**	**-0.15**	**-0.22**	**-0.22**			
Tanzania	0.76	0.75		**-0.15**	**-0.23**	**-0.17**				
Zambia	0.72	0.35		-0.08	**-0.18**	-0.06	**-0.14**			
Democratic Republic of Congo	0.31	0.56		-0.03	**-0.11**					
Cameroon	0.29	0.36		-0.12	-0.01	**-0.25**				
Côte d'Ivoire	0.14	0.33		-0.02	-0.27	-0.12				
Niger	0.14	0.37		-0.07	0.01	0.02				
Burkina Faso	0.13	0.55		-0.08	-0.11	**-0.23**	-0.02			
Chad	0.07	0.60		-0.07	-0.14					
Mozambique	-0.25	0.35		-0.05	-0.08	**-0.15**				
Mali	-1.07	0.25		-0.01	-0.08	-0.09	-0.13			

Legend: Countries are ranked by annual percentage changes in HAZ between the first and last survey. †P associated with temporal stability test of household wealth between the first and last survey from the flexible model. For countries that had $P < .05$ in the flexible model, the parsimonious model showed linearly increasing (yellow) or decreasing (blue) association or no linear trend in the association (grey) of lack of household wealth (defined as belonging to the poorest wealth quintile) with HAZ over survey rounds. ‡Estimated associations by survey round from flexible model. Coefficients significant (5% level) are indicated in bold. Abbreviations: HAZ, height-for-age z-score.

Appendix Table 8. Results of flexible and parsimonious models for child's gender in Demographic and Health Surveys in 25 countries

Country	Annual change in HAZ in %	Equality of associations between first and last survey round (P) †	Association between HAZ and child being a girl across survey rounds ‡							
			Trend of associations	Survey						
				1	2	3	4	5	6	7
Turkey	4.71	0.47		0.00	0.00	-0.04				
Egypt	3.61	0.29		**0.11**	**0.12**	**0.17**	**0.18**	**0.16**		
Ethiopia	2.59	0.82		**0.09**	**0.18**	**0.08**				
Bangladesh	2.22	0.40		0.05	0.02	0.03	-0.04	0.01		
Cambodia	2.10	0.94		0.06	**0.10**	0.00	0.06			
Peru	1.88	0.02		**0.12**	**0.13**	**0.05**	**0.09**	**0.08**	**0.04**	**0.05**
Nepal	1.64	0.54		**0.11**	0.00	0.03	0.07			
Ghana	1.56	0.54		**0.16**	**0.16**	**0.26**	0.10	**0.12**		
Kenya	1.52	0.82		**0.19**	**0.26**	**0.28**	**0.19**	**0.20**		
Madagascar	1.35	0.05		**0.35**	**0.26**	**0.22**				
Nigeria	1.32	0.38		**0.25**	**0.23**	**0.20**				
India	1.30	0.12		**0.09**	**0.05**					
Uganda	1.16	0.49		**0.29**	**0.16**	**0.16**	**0.24**			
Rwanda	1.06	0.76		**0.23**	**0.15**	**0.21**				
Malawi	1.01	0.90		**0.25**	**0.19**	**0.23**	**0.24**			
Tanzania	0.76	0.66		**0.21**	**0.18**	**0.18**				
Zambia	0.72	0.92		**0.20**	**0.22**	**0.27**	**0.19**			
Democratic Republic of Congo	0.31	0.78		**0.23**	**0.25**					
Cameroon	0.29	0.04		**0.31**	0.07	**0.13**				
Côte d'Ivoire	0.14	0.84		**0.20**	0.12	**0.21**				
Niger	0.14	0.92		**0.15**	**0.18**	**0.16**				
Burkina Faso	0.13	0.64		**0.14**	**0.17**	**0.22**	**0.17**			
Chad	0.07	0.31		0.06	**0.13**					
Mozambique	-0.25	0.10		**0.31**	**0.17**	**0.20**				
Mali	-1.07	0.64		**0.14**	**0.13**	**0.15**	**0.11**			

Legend: Countries are ranked by annual percentage changes in HAZ between the first and last survey. †P associated with temporal stability test of child's gender between the first and last survey from the flexible model. For countries that had P < .05 in the flexible model, the parsimonious model showed linearly decreasing association (blue) or no linear trend in the association (grey) of child being a girl with HAZ over survey rounds. ‡Estimated associations by survey round from flexible model. Coefficients significant (5% level) are indicated in bold. Abbreviations: HAZ, height-for-age z-score.

Appendix Table 9. Results of flexible and parsimonious models for child's birth order in Demographic and Health Surveys in 25 countries

Country	Annual change in HAZ in %	Equality of associations between first and last survey round (P) †	Association between HAZ and being firstborn across survey rounds ‡							
			Trend of associations	Survey						
				1	2	3	4	5	6	7
Turkey	4.71	0.89	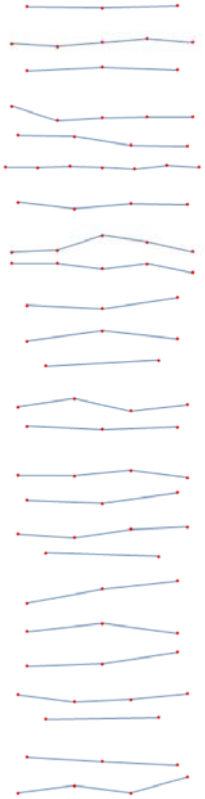	**0.13**	**0.12**	**0.14**				
Egypt	3.61	0.73		0.07	0.03	**0.08**	**0.13**	**0.09**		
Ethiopia	2.59	0.83		**0.13**	**0.18**	**0.15**				
Bangladesh	2.22	0.04		0.09	**-0.10**	-0.07	-0.05	-0.05		
Cambodia	2.10	0.13		0.13	**0.12**	0.00	-0.02			
Peru	1.88	0.97		**0.14**	**0.13**	**0.15**	**0.13**	**0.11**	**0.16**	**0.13**
Nepal	1.64	0.73		0.09	0.00	0.07	0.06			
Ghana	1.56	0.92		-0.15	-0.12	0.08	-0.01	**-0.14**		
Kenya	1.52	0.10		**0.14**	0.14	0.06	**0.13**	0.01		
Madagascar	1.35	0.33		0.01	-0.04	0.11				
Nigeria	1.32	0.73		-0.05	**0.09**	-0.02				
India	1.30	0.02		0.05	**0.13**					
Uganda	1.16	0.79		-0.07	0.04	-0.12	-0.04			
Rwanda	1.06	0.90		0.10	0.06	0.09				
Malawi	1.01	0.71		-0.12	**-0.13**	-0.06	**-0.16**			
Tanzania	0.76	0.16		-0.03	-0.07	0.07				
Zambia	0.72	0.20		-0.05	-0.10	0.01	0.04			
Democratic Republic of Congo	0.31	0.66		0.13	0.08					
Cameroon	0.29	0.02		-0.12	0.07	**0.19**				
Côte d'Ivoire	0.14	0.82		-0.06	0.04	-0.09				
Niger	0.14	0.07		-0.10	-0.07	0.08				
Burkina Faso	0.13	0.92		-0.05	-0.15	**-0.12**	-0.04			
Chad	0.07	0.84		0.05	0.07					
Mozambique	-0.25	0.26		-0.03	-0.09	**-0.14**				
Mali	-1.07	0.06		-0.08	0.01	-0.08	0.13			

Legend: Countries are ranked by annual percentage changes in HAZ between the first and last survey. †P associated with temporal stability test of child's birth order between the first and last survey from the flexible model. For countries that had P < .05 in the flexible model, the parsimonious model showed linearly increasing association (yellow) or no linear trend in the association (grey) of child being firstborn with HAZ over survey rounds. ‡Estimated associations by survey round from flexible model. Coefficients significant (5% level) are indicated in bold. Abbreviations: HAZ, height-for-age z-score.

Appendix Table 10. Results of flexible and parsimonious models for mortality of under-5 children in Demographic and Health Surveys in 25 countries

Country	Annual change in HAZ in %	Equality of associations between first and last survey round (P) †	Association between HAZ and under-5 mortality in the village across survey rounds ‡							
			Trend of associations	Survey						
				1	2	3	4	5	6	7
Turkey	4.71	0.80	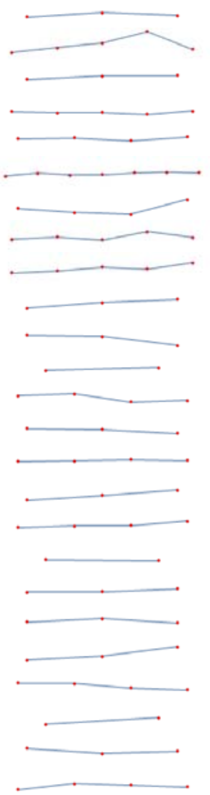	**-0.85**	-0.27	-0.69				
Egypt	3.61	0.52		**-1.31**	-0.65	-0.02	**1.56**	-0.86		
Ethiopia	2.59	0.28		**-0.60**	-0.17	-0.11				
Bangladesh	2.22	0.89		-0.59	**-0.78**	**-0.74**	**-0.99**	-0.53		
Cambodia	2.10	0.64		0.02	0.17	-0.22	0.29			
Peru	1.88	0.29		**-0.66**	-0.30	**-0.56**	-0.48	-0.21	-0.18	-0.24
Nepal	1.64	0.12		-0.73	**-1.29**	**-1.53**	0.48			
Ghana	1.56	0.73		-0.51	-0.20	-0.59	0.48	-0.31		
Kenya	1.52	0.00		**-0.72**	-0.51	0.04	-0.21	**0.62**		
Madagascar	1.35	0.13		**-1.18**	-0.42	0.00				
Nigeria	1.32	0.01		-0.46	-0.65	**-1.95**				
India	1.30	0.48		-0.92	**-0.57**					
Uganda	1.16	0.46		-0.06	0.20	-0.84	-0.65			
Rwanda	1.06	0.22		-0.07	-0.21	-0.73				
Malawi	1.01	0.86		-0.21	-0.13	0.05	-0.12			
Tanzania	0.76	0.01		**-1.10**	-0.50	0.19				
Zambia	0.72	0.09		-0.47	-0.30	-0.24	0.34			
Democratic Republic of Congo	0.31	0.85		-0.45	-0.59					
Cameroon	0.29	0.49		-0.53	-0.50	-0.08				
Côte d'Ivoire	0.14	0.78		-0.21	0.24	-0.38				
Niger	0.14	0.01		**-1.05**	-0.53	0.74				
Burkina Faso	0.13	0.15		0.14	0.07	-0.47	-0.74			
Chad	0.07	0.18		**-1.02**	-0.14					
Mozambique	-0.25	0.50		0.00	**-0.68**	-0.35				
Mali	-1.07	0.63		**-1.23**	-0.42	-0.61	-0.86			

Legend: Countries are ranked by annual percentage changes in HAZ between the first and last survey. †P associated with temporal stability test of mortality of under-5 children between the first and last survey from the flexible model. For countries that had P < .05 in the flexible model, the parsimonious model showed linearly increasing (yellow) or decreasing (blue) association of mortality of under-5 children with HAZ over survey rounds. ‡Estimated associations by survey round from flexible model. Coefficients significant (5% level) are indicated in bold. Abbreviations: HAZ, height-for-age z-score.

Appendix Table 11. Results of flexible and parsimonious models for access to sanitation in Demographic and Health Surveys in 25 countries

Country	Annual change in HAZ in %	Equality of associations between first and last survey round (P) †	Association between HAZ and no sanitation (village average) across survey rounds ‡							
			Trend of associations	Survey						
				1	2	3	4	5	6	7
Turkey	4.71	0.55		-0.39	0.00	**-0.87**				
Egypt	3.61	0.01		**-0.57**	**-0.56**	-0.88	1.81	**1.78**		
Ethiopia	2.59	0.00		**-0.37**	0.22	0.00				
Bangladesh	2.22	0.02		**0.30**	0.13	-0.01	-0.19	-0.15		
Cambodia	2.10	0.92		**-0.42**	**-0.61**	**-0.29**	**-0.44**			
Peru	1.88	0.17		**-0.43**	**-0.42**	**-0.31**	**-0.26**	**-0.26**	**-0.18**	**-0.29**
Nepal	1.64	0.91		**-0.30**	-0.11	-0.17	**-0.28**			
Ghana	1.56	0.12		**-0.40**	**-0.26**	**-0.25**	-0.12	-0.17		
Kenya	1.52	0.05		**-0.25**	0.01	0.03	0.03	0.01		
Madagascar	1.35	0.17		**0.33**	0.23	**0.54**				
Nigeria	1.32	0.18		-0.07	**-0.15**	0.12				
India	1.30	0.00		0.09	**-0.35**					
Uganda	1.16	0.19		-0.08	-0.09	**-0.32**	0.25			
Rwanda	1.06	0.59		0.12	-0.65	-0.35				
Malawi	1.01	0.84		0.02	-0.01	-0.19	-0.03			
Tanzania	0.76	0.84		-0.05	0.16	-0.02				
Zambia	0.72	0.00		**0.28**	0.12	**0.25**	-0.08			
Democratic Republic of Congo	0.31	0.13		0.25	-0.13					
Cameroon	0.29	0.77		-0.11	-0.18	-0.02				
Côte d'Ivoire	0.14	0.44		-0.09	-0.24	**-0.22**				
Niger	0.14	0.48		**-0.31**	-0.08	**-0.45**				
Burkina Faso	0.13	0.08		**-0.57**	-0.05	**-0.27**	**-0.22**			
Chad	0.07	0.03		0.01	**-0.50**					
Mozambique	-0.25	0.23		**-0.24**	-0.01	-0.08				
Mali	-1.07	0.27		-0.13	**-0.31**	**-0.37**	**-0.36**			

Legend: Countries are ranked by annual percentage changes in HAZ between the first and last survey. †P associated with temporal stability test of access to sanitation between the first and last survey from the flexible model. For countries that had P < .05 in the flexible model, the parsimonious model showed linearly increasing (yellow) or decreasing (blue) association or no linear trend in the association (grey) of lack of access to sanitation (defined as having no toilet) with HAZ over survey rounds. ‡Estimated associations by survey round from flexible model. Coefficients significant (5% level) are indicated in bold. Abbreviations: HAZ, height-for-age z-score.

Appendix Table 12. Results of flexible and parsimonious models for age larger 21 months in Demographic and Health Surveys in 25 countries

Country	Annual change in HAZ in %	Equality of associations between first and last survey round (P) †	Association between HAZ and child being older than 21 months across survey rounds ‡							
			Trend of associations	Survey						
				1	2	3	4	5	6	7
Turkey	4.71	0.73		**-1.43**	**-1.57**	**-1.35**				
Egypt	3.61	0.00		**-2.22**	**-1.10**	**-0.88**	**-1.46**	**-0.74**		
Ethiopia	2.59	0.02		**-2.56**	**-2.50**	**-2.90**				
Bangladesh	2.22	0.02		**-1.90**	**-1.52**	**-1.47**	**-1.58**	**-1.51**		
Cambodia	2.10	0.03		**-1.70**	**-1.64**	**-1.54**	**-1.26**			
Peru	1.88	0.00		**-1.57**	**-1.46**	**-1.60**	**-1.16**	**-0.78**	**-0.81**	**-0.67**
Nepal	1.64	0.48		**-1.46**	**-1.90**	**-1.83**	**-1.71**			
Ghana	1.56	0.08		**-1.05**	**-2.23**	**-2.71**	**-2.43**	**-1.83**		
Kenya	1.52	0.00		**-2.14**	**-1.46**	**-2.42**	**-2.23**	**-1.64**		
Madagascar	1.35	0.45		**-1.57**	**-1.63**	**-1.27**				
Nigeria	1.32	0.50		**-2.37**	**-1.99**	**-2.25**				
India	1.30	0.00		**-1.05**	**-1.87**					
Uganda	1.16	0.04		**-1.76**	**-1.79**	**-1.75**	**-2.28**			
Rwanda	1.06	0.36		**-1.99**	**-2.12**	**-1.83**				
Malawi	1.01	0.03		**-2.30**	**-2.41**	**-2.15**	**-1.87**			
Tanzania	0.76	0.97		**-2.13**	**-1.83**	**-2.13**				
Zambia	0.72	0.34		**-2.28**	**-2.44**	**-2.07**	**-2.15**			
Democratic Republic of Congo	0.31	0.44		**-1.86**	**-1.67**					
Cameroon	0.29	0.07		**-1.67**	**-2.14**	**-2.39**				
Côte d'Ivoire	0.14	0.03		**-1.15**	**-1.42**	**-1.83**				
Niger	0.14	0.19		**-1.97**	**-3.19**	**-2.40**				
Burkina Faso	0.13	0.01		**-2.92**	**-2.87**	**-3.11**	**-2.48**			
Chad	0.07	0.40		**-3.33**	**-3.50**					
Mozambique	-0.25	0.61		**-1.38**	**-2.03**	**-1.56**				
Mali	-1.07	0.00		**-1.49**	**-2.75**	**-2.72**	**-2.59**			

Legend: Countries are ranked by annual percentage changes in HAZ between the first and last survey. †P associated with temporal stability test of age larger 21 months between the first and last survey from the flexible model. For countries that had P < .05 in the flexible model, the parsimonious model showed linearly increasing (yellow) or decreasing (blue) association or no linear trend in the association (grey) of age larger 21 months with HAZ over survey rounds. In addition, Madagascar, Mozambique, and Nepal showed a linearly increasing association in the parsimonious model, despite having P > .05 in the flexible model. ‡Estimated associations by survey round from flexible model. Coefficients significant (5% level) are indicated in bold. Abbreviations: HAZ, height-for-age z-score.

Appendix Figure 1. Temporal stability of covariates versus of growth faltering in 25 countries*

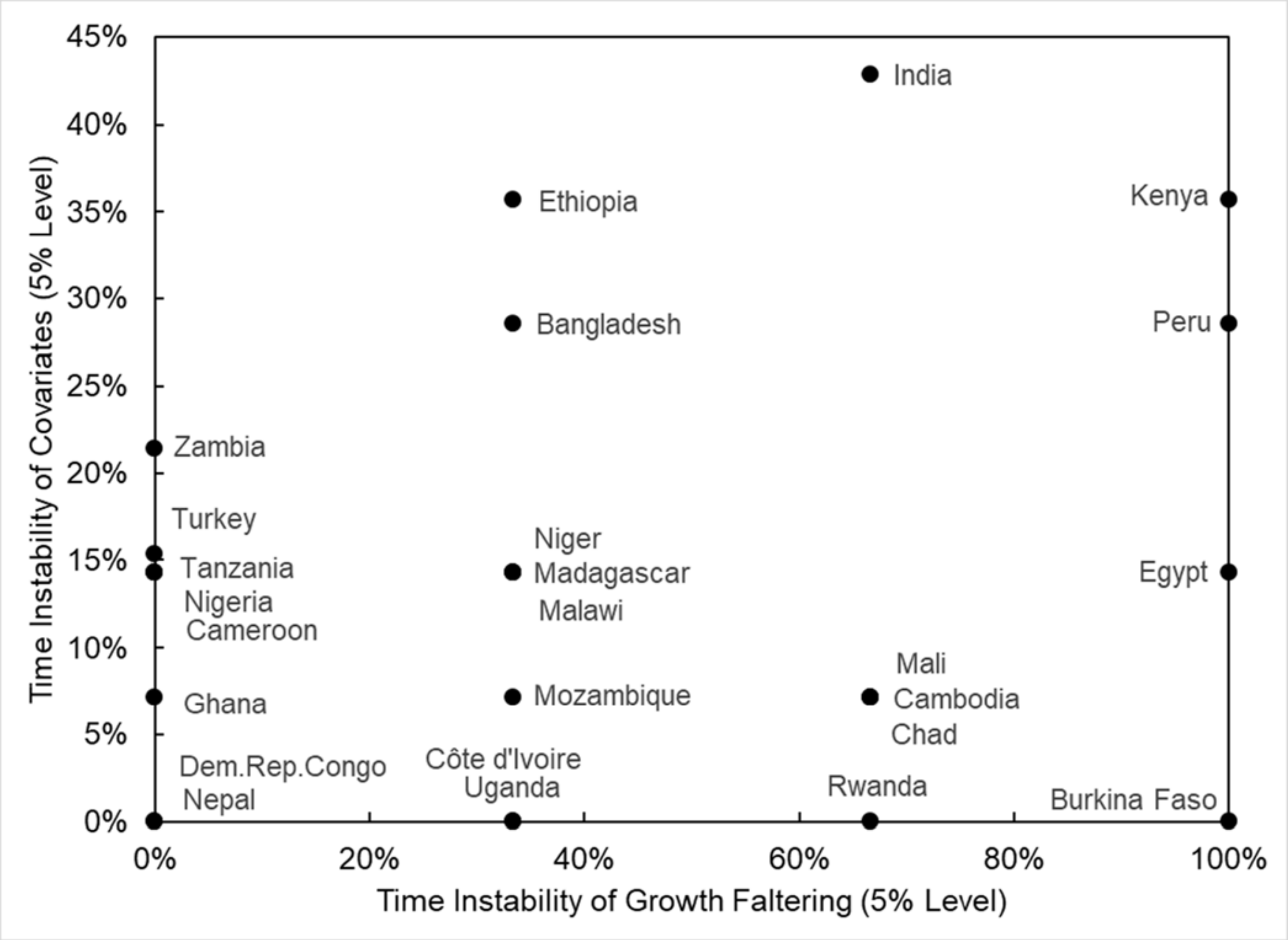

*Plot of % covariates with significantly unstable coefficients between the first and last survey for each country as indicated by Wald test versus % growth faltering variables with significantly unstable coefficients as indicated by Wald test (5% level).

Appendix Figure 2. Alternative ranking of countries by the temporal stability of their coefficients in 25 countries*

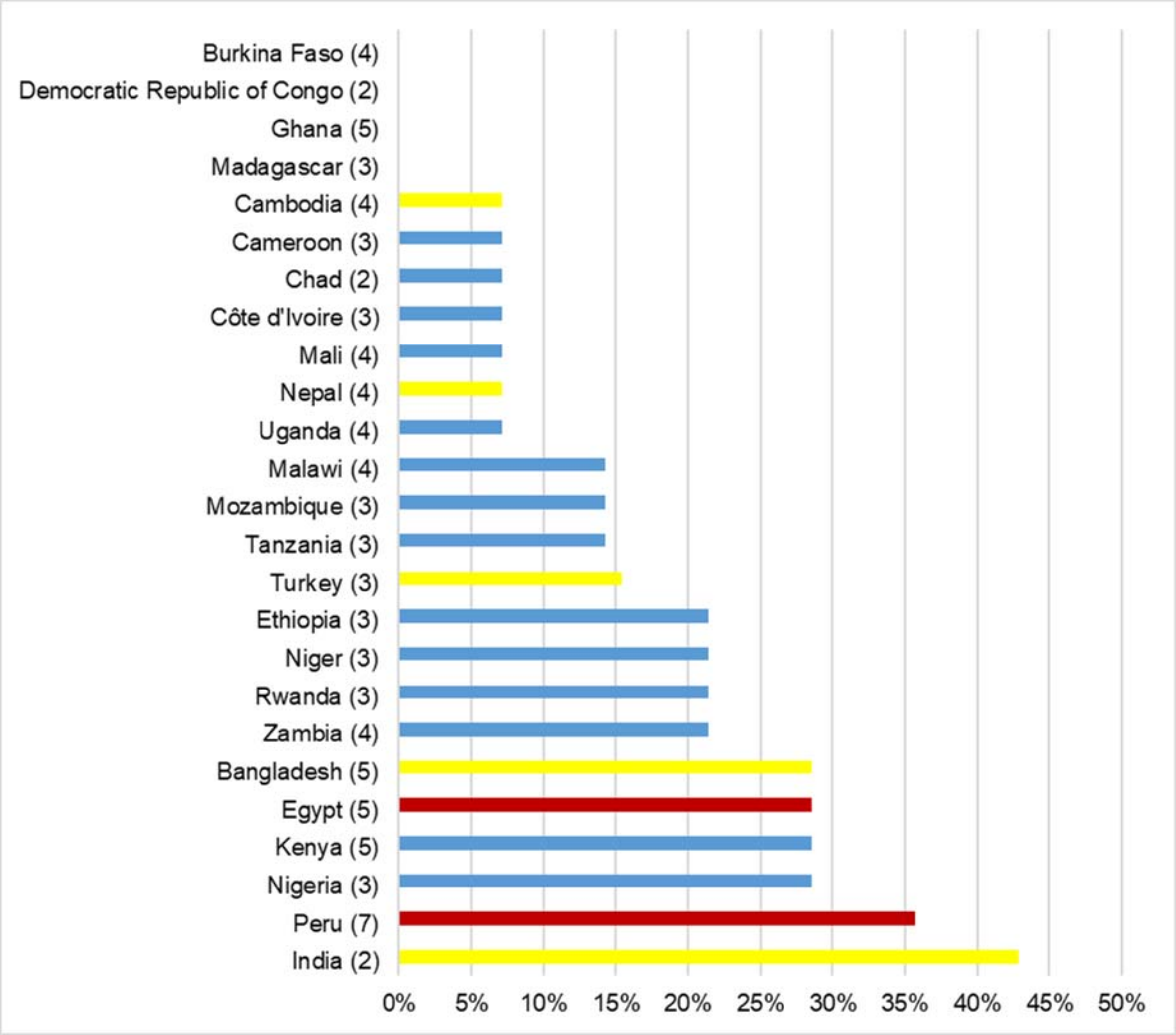

*Plot of % covariates with significantly unstable coefficients across *all* surveys in each country as indicated by Wald test (5% level), excluding growth faltering variables. Number of survey rounds for each country is displayed in parentheses. Country groups: yellow, Asia; blue, Sub-Saharan Africa; red, other.

Appendix Figure 3. Time between the first and last Demographic and Health Survey versus number of survey rounds and time instability of covariates in 25 countries*

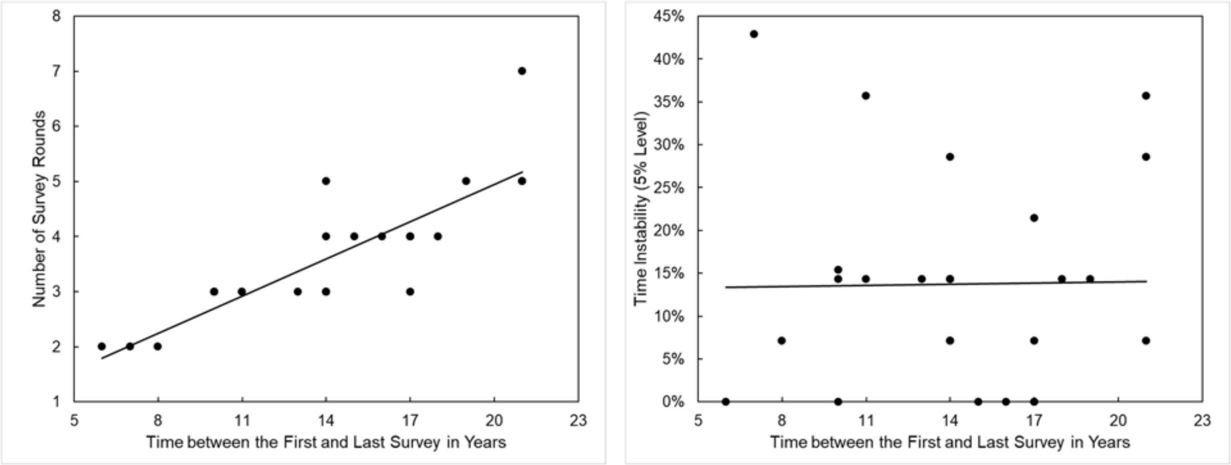

*Each country represents a point in each graph. Y-axes: left, number of Demographic and Health Survey rounds; right, % covariates with significantly unstable coefficients between the first and last survey as indicated by Wald test in each country (5% significance level), excluding growth faltering variables. X-axes: time difference between the first and last Demographic and Health Survey round in years.

Appendix Figure 4. Temporal stability of covariates in WHZ regressions in Demographic and Health Surveys in 25 countries

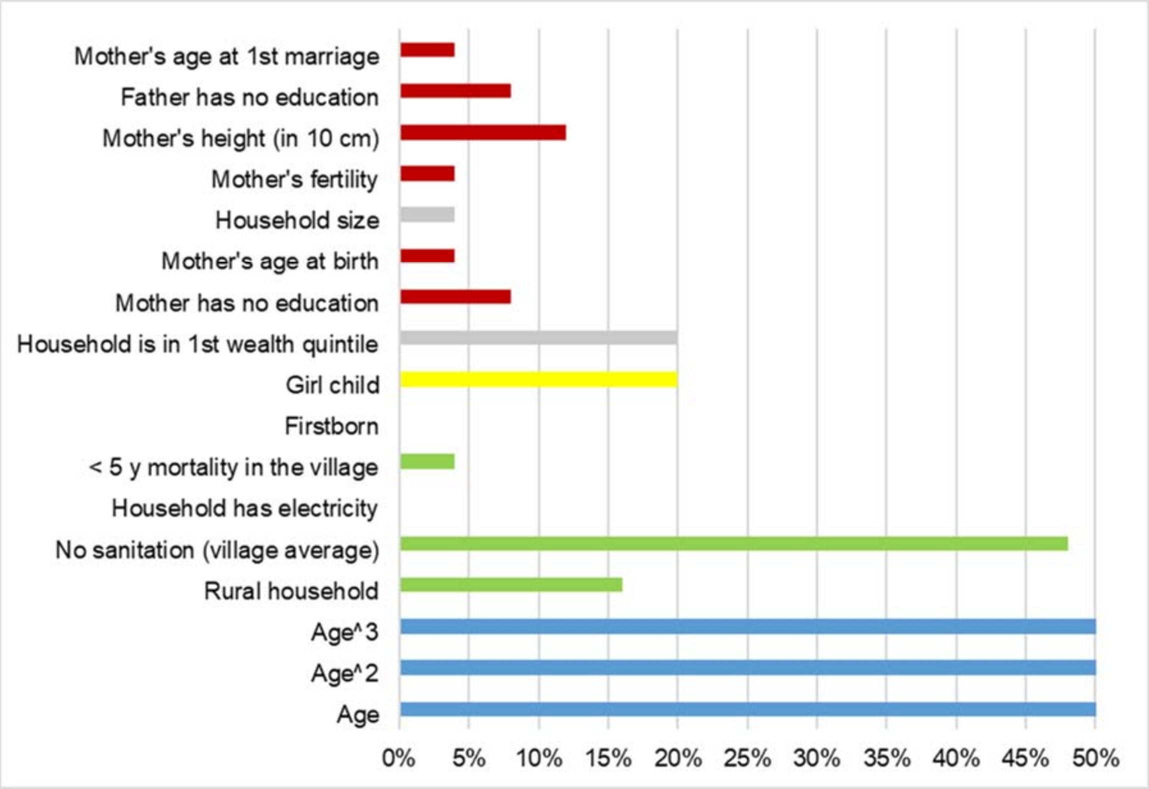

Legend: Data reported as % countries in which the coefficient associated with the covariate was significantly unstable in WHZ regressions between the first and last survey as indicated by Wald test (5% significance level). Variable groups: yellow, child; red, parental; grey, household; green, community and infrastructure; blue, age-related variables. There was no apparent grouping of variables in terms of temporal stability.
