# Supplementary material for: Temporal stability of child growth associations in Demographic and Health Surveys in 25 countries
Source: SSM Popul Health. 2019 Jan 12;7:100352. doi: 10.1016/j.ssmph.2019.100352 (PMC6351606; doi:10.1016/j.ssmph.2019.100352)
Supplement: Supplementary file 2 — Supplementary material [file mmc2.pdf]

**Appendix Table 2. Associations between determinants of child growth and HAZ in different survey rounds in flexible models in Demographic and Health Surveys in 25 countries**

| Country | 1<br>Bangladesh | 2<br>Burkina Faso | 3<br>Cambodia | 4<br>Cameroon | 5<br>Chad | 6<br>Democratic Republic of Congo | 7<br>Côte d'Ivoire | 8<br>Egypt | 9<br>Ethiopia | 10<br>Ghana | 11<br>India | 12<br>Kenya | 13<br>Madagascar | 14<br>Malawi | 15<br>Mali | 16<br>Mozambique | 17<br>Nepal | 18<br>Niger | 19<br>Nigeria | 20<br>Peru | 21<br>Rwanda | 22<br>Tanzania | 23<br>Turkey | 24<br>Uganda | 25<br>Zambia |
|---|---|---|---|---|---|---|---|---|---|---|---|---|---|---|---|---|---|---|---|---|---|---|---|---|---|
| **Survey year** | **1997, 2000, 2004, 2007, 2011** | **1993, 1998, 2003, 2010** | **2000, 2005, 2010, 2014** | **1998, 2004, 2011** | **1996, 2004** | **2007, 2013** | **1994, 1998, 2011** | **1995, 2000, 2005, 2008, 2014** | **2000, 2005, 2011** | **1993, 1998, 2003, 2008, 2014** | **1998, 2005** | **1993, 1998, 2003, 2008, 2014** | **1997, 2003, 2008** | **1992, 2000, 2004, 2010** | **1995, 2001, 2006, 2012** | **1997, 2003, 2011** | **1996, 2001, 2006, 2011** | **1998, 2006, 2012** | **2003, 2008, 2013** | **1991, 1996, 2000, 2006, 2010, 2011, 2012** | **2000, 2005, 2010** | **1996, 2004, 2010** | **1993, 1998, 2003** | **1995, 2000, 2006, 2011** | **1996, 2001, 2007, 2013** |
| ***1. Regression results*** | | | | | | | | | | | | | | | | | | | | | | | | | |
| ***Growth faltering indicators*** | | | | | | | | | | | | | | | | | | | | | | | | | |
| Age (in months)_1 | -0.0865*** | -0.1235*** | -0.0854*** | -0.1096*** | -0.1365*** | -0.0880*** | -0.0793*** | -0.0922*** | -0.1246*** | -0.0797*** | -0.0922*** | -0.0933*** | -0.0948*** | -0.0893*** | -0.1130*** | -0.0927*** | -0.0794*** | -0.1230*** | -0.1100*** | -0.0721*** | -0.1152*** | -0.0810*** | -0.0526*** | -0.0743*** | -0.0934*** |
| | (0.0052) | (0.0056) | (0.0084) | (0.0071) | (0.0063) | (0.0103) | (0.0060) | (0.0047) | (0.0053) | (0.0072) | (0.0021) | (0.0060) | (0.0047) | (0.0068) | (0.0047) | (0.0065) | (0.0038) | (0.0048) | (0.0073) | (0.0043) | (0.0059) | (0.0055) | (0.0043) | (0.0051) | (0.0050) |
| Age (in months)_2 | -0.0611*** | -0.1230*** | -0.0852*** | -0.0973*** | -0.1660*** | -0.0753*** | -0.0604*** | -0.0292*** | -0.1268*** | -0.0975*** | -0.0938*** | -0.0939*** | -0.0750*** | -0.1062*** | -0.1186*** | -0.0809*** | -0.0747*** | -0.1377*** | -0.1081*** | -0.0711*** | -0.1053*** | -0.0748*** | -0.0662*** | -0.0821*** | -0.1041*** |
| | (0.0040) | (0.0077) | (0.0056) | (0.0082) | (0.0089) | (0.0053) | (0.0129) | (0.0049) | (0.0091) | (0.0070) | (0.0022) | (0.0067) | (0.0074) | (0.0046) | (0.0044) | (0.0045) | (0.0042) | (0.0072) | (0.0048) | (0.0034) | (0.0068) | (0.0043) | (0.0047) | (0.0050) | (0.0055) |
| Age (in months)_3 | -0.0627*** | -0.1503*** | -0.0715*** | -0.0957*** | | | -0.0746*** | -0.0229*** | -0.1256*** | -0.1125*** | | -0.0917*** | -0.0720*** | -0.1094*** | -0.1271*** | -0.0677*** | -0.0820*** | -0.0866*** | -0.0970*** | -0.0684*** | -0.0839*** | -0.0913*** | -0.0436*** | -0.0789*** | -0.1049*** |
| | (0.0041) | (0.0056) | (0.0069) | (0.0066) | | | (0.0068) | (0.0054) | (0.0049) | (0.0079) | | (0.0064) | (0.0077) | (0.0052) | (0.0047) | (0.0047) | (0.0042) | (0.0066) | (0.0036) | (0.0037) | (0.0068) | (0.0050) | (0.0040) | (0.0087) | (0.0073) |
| Age (in months)_4 | -0.0588*** | -0.1016*** | -0.0621*** | | | | | -0.0673*** | | -0.1097*** | | -0.0988*** | | -0.0949*** | -0.1014*** | | -0.0651*** | | | -0.0563*** | | | | -0.0867*** | -0.0893*** |
| | (0.0047) | (0.0052) | (0.0062) | | | | | (0.0052) | | (0.0096) | | (0.0066) | | (0.0065) | (0.0088) | | (0.0072) | | | (0.0032) | | | | (0.0088) | (0.0045) |
| Age (in months)_5 | -0.0767*** | | | | | | | -0.0311*** | | -0.0618*** | | -0.0693*** | | | | | | | | -0.0392*** | | | | | |
| | (0.0045) | | | | | | | (0.0048) | | (0.0074) | | (0.0043) | | | | | | | | (0.0033) | | | | | |
| Age (in months)_6 | | | | | | | | | | | | | | | | | | | | -0.0378*** | | | | | |
| | | | | | | | | | | | | | | | | | | | | (0.0033) | | | | | |
| Age (in months)_7 | | | | | | | | | | | | | | | | | | | | -0.0344*** | | | | | |
| | | | | | | | | | | | | | | | | | | | | (0.0030) | | | | | |
| Older than 21 months_1 | -1.9018*** | -2.9235*** | -1.7040*** | -1.6748*** | -3.3285*** | -1.8636*** | -1.1527*** | -2.2245*** | -2.5560*** | -1.0534** | -1.0513*** | -2.1428*** | -1.5693*** | -2.3001*** | -1.4913*** | -1.3772*** | -1.4575*** | -1.9661*** | -2.3663*** | -1.5683*** | -1.9939*** | -2.1315*** | -1.4297*** | -1.7574*** | -2.2781*** |
| | (0.1281) | (0.1329) | (0.1559) | (0.3786) | (0.1281) | (0.2215) | (0.2842) | (0.0962) | (0.1057) | (0.4254) | (0.1542) | (0.1160) | (0.3645) | (0.1401) | (0.2611) | (0.3416) | (0.3310) | (0.3105) | (0.1514) | (0.0812) | (0.1243) | (0.1121) | (0.1750) | (0.1699) | (0.1033) |
| Older than 21 months_2 | -1.5159*** | -2.8679*** | -1.6350*** | -2.1388*** | -3.5027*** | -1.6712*** | -1.4183*** | -1.0950*** | -2.5042*** | -2.2323*** | -1.8656*** | -1.4622*** | -1.6251*** | -2.4054*** | -2.7505*** | -2.0306*** | -1.9016*** | -3.1917*** | -1.9939*** | -1.4626*** | -2.1201*** | -1.8287*** | -1.5729*** | -1.7900*** | -2.4358*** |
| | (0.1032) | (0.1630) | (0.1154) | (0.1691) | (0.1626) | (0.1146) | (0.3134) | (0.1027) | (0.1719) | (0.1587) | (0.0484) | (0.3479) | (0.1632) | (0.0940) | (0.1138) | (0.0916) | (0.0934) | (0.1500) | (0.0941) | (0.0632) | (0.1310) | (0.0850) | (0.1699) | (0.1090) | (0.1176) |
| Older than 21 months_3 | -1.4694*** | -3.1124*** | -1.5371*** | -2.3892*** | | | -1.8308*** | -0.8787*** | -2.8965*** | -2.7108*** | | -2.4193*** | -1.2706*** | -2.1533*** | -2.7177*** | -1.5600*** | -1.8252*** | -2.4026*** | -2.2523*** | -1.6011*** | -1.8329*** | -2.1264*** | -1.3545*** | -1.7476*** | -2.0732*** |
| | (0.0967) | (0.1179) | (0.1291) | (0.1216) | | | (0.1314) | (0.1066) | (0.1015) | (0.1404) | | (0.1292) | (0.1510) | (0.1055) | (0.1106) | (0.1000) | (0.0868) | (0.1284) | (0.0739) | (0.0655) | (0.1222) | (0.0938) | (0.1335) | (0.1804) | (0.1300) |
| Older than 21 months_4 | -1.5825*** | -2.4805*** | -1.2606*** | | | | | -1.4633*** | | -2.4336*** | | -2.2321*** | | -1.8726*** | -2.5884*** | | -1.7149*** | | | -1.1588*** | | | | -2.2822*** | -2.1503*** |
| | (0.1057) | (0.1113) | (0.1287) | | | | | (0.1207) | | (0.1786) | | (0.1261) | | (0.1315) | (0.1650) | | (0.1466) | | | (0.0633) | | | | (0.1846) | (0.0845) |
| Older than 21 months_5 | -1.5111*** | | | | | | | -0.7387*** | | -1.8314*** | | -1.6376*** | | | | | | | | -0.7751*** | | | | | |
| | (0.0985) | | | | | | | (0.1033) | | (0.1366) | | (0.0853) | | | | | | | | (0.0597) | | | | | |
| Older than 21 months_6 | | | | | | | | | | | | | | | | | | | | -0.8124*** | | | | | |
| | | | | | | | | | | | | | | | | | | | | (0.0584) | | | | | |
| Older than 21 months_7 | | | | | | | | | | | | | | | | | | | | -0.6679*** | | | | | |
| | | | | | | | | | | | | | | | | | | | | (0.0575) | | | | | |
| Age (in months) * Older than 21 months_1 | 0.0915*** | 0.1369*** | 0.0801*** | 0.0946*** | 0.1530*** | 0.0777*** | 0.0553*** | 0.1113*** | 0.1314*** | 0.0452*** | 0.0628*** | 0.1047*** | 0.0842*** | 0.0979*** | 0.0757*** | 0.0713*** | 0.0669*** | 0.1054*** | 0.1215*** | 0.0746*** | 0.1126*** | 0.0946*** | 0.0527*** | 0.0824*** | 0.1044*** |
| | (0.0056) | (0.0062) | (0.0088) | (0.0144) | (0.0067) | (0.0110) | (0.0111) | (0.0049) | (0.0057) | (0.0159) | (0.0053) | (0.0061) | (0.0128) | (0.0072) | (0.0098) | (0.0131) | (0.0112) | (0.0115) | (0.0078) | (0.0046) | (0.0062) | (0.0058) | (0.0056) | (0.0064) | (0.0052) |
| Age (in months) * Older than 21 months_2 | 0.0695*** | 0.1344*** | 0.0829*** | 0.1081*** | 0.1791*** | 0.0693*** | 0.0638*** | 0.0422*** | 0.1305*** | 0.1020*** | 0.0982*** | 0.0838*** | 0.0807*** | 0.1147*** | 0.1328*** | 0.0949*** | 0.0817*** | 0.1556*** | 0.1132*** | 0.0737*** | 0.1103*** | 0.0849*** | 0.0664*** | 0.0905*** | 0.1172*** |
| | (0.0043) | (0.0080) | (0.0059) | (0.0087) | (0.0092) | (0.0058) | (0.0151) | (0.0053) | (0.0093) | (0.0077) | (0.0023) | (0.0136) | (0.0080) | (0.0049) | (0.0049) | (0.0047) | (0.0043) | (0.0075) | (0.0051) | (0.0036) | (0.0073) | (0.0045) | (0.0058) | (0.0056) | (0.0059) |
| Age (in months) * Older than 21 months_3 | 0.0682*** | 0.1588*** | 0.0711*** | 0.1059*** | | | 0.0802*** | 0.0330*** | 0.1353*** | 0.1272*** | | 0.1111*** | 0.0666*** | 0.1168*** | 0.1403*** | 0.0766*** | 0.0841*** | 0.1060*** | 0.1052*** | 0.0742*** | 0.0898*** | 0.1051*** | 0.0452*** | 0.0847*** | 0.1107*** |
| | (0.0045) | (0.0059) | (0.0072) | (0.0067) | | | (0.0070) | (0.0058) | (0.0054) | (0.0079) | | (0.0068) | (0.0080) | (0.0053) | (0.0052) | (0.0051) | (0.0043) | (0.0071) | (0.0039) | (0.0038) | (0.0071) | (0.0051) | (0.0048) | (0.0092) | (0.0075) |
| Age (in months) * Older than 21 months_4 | 0.0648*** | 0.1153*** | 0.0602*** | | | | | 0.0728*** | | 0.1150*** | | 0.1123*** | | 0.0998*** | 0.1123*** | | 0.0701*** | | | 0.0623*** | | | | 0.1040*** | 0.1053*** |
| | (0.0049) | (0.0055) | (0.0066) | | | | | (0.0057) | | (0.0102) | | (0.0068) | | (0.0067) | (0.0090) | | (0.0073) | | | (0.0033) | | | | (0.0095) | (0.0047) |
| Age (in months) * Older than 21 months_5 | 0.0828*** | | | | | | | 0.0327*** | | 0.0741*** | | 0.0792*** | | | | | | | | 0.0439*** | | | | | |
| | (0.0048) | | | | | | | (0.0051) | | (0.0076) | | (0.0046) | | | | | | | | (0.0034) | | | | | |
| Age (in months) * Older than 21 months_6 | | | | | | | | | | | | | | | | | | | | 0.0445*** | | | | | |
| | | | | | | | | | | | | | | | | | | | | (0.0034) | | | | | |
| Age (in months) * Older than 21 months_7 | | | | | | | | | | | | | | | | | | | | 0.0387*** | | | | | |
| | | | | | | | | | | | | | | | | | | | | (0.0032) | | | | | |
| ***Child's characteristics*** | | | | | | | | | | | | | | | | | | | | | | | | | |
| Girl child_1 | 0.0545 | 0.1404*** | 0.0597 | 0.3051*** | 0.0572 | 0.2281*** | 0.1964*** | 0.1108*** | 0.0928*** | 0.1634** | 0.0866*** | 0.1922*** | 0.3526*** | 0.2491*** | 0.1447*** | 0.3117*** | 0.1117*** | 0.1543*** | 0.2469*** | 0.1208*** | 0.2303*** | 0.2061*** | 0.0034 | 0.2863*** | 0.1993*** |
| | (0.0404) | (0.0490) | (0.0520) | (0.0745) | (0.0454) | (0.0660) | (0.0490) | (0.0307) | (0.0328) | (0.0651) | (0.0208) | (0.0442) | (0.0515) | (0.0436) | (0.0434) | (0.0579) | (0.0403) | (0.0435) | (0.0547) | (0.0264) | (0.0410) | (0.0364) | (0.0468) | (0.0392) | (0.0382) |
| Girl child_2 | 0.0200 | 0.1685*** | 0.1031*** | 0.0707 | 0.1265** | 0.2493*** | 0.1232 | 0.1224*** | 0.1828*** | 0.1624*** | 0.0465*** | 0.2645*** | 0.2577*** | 0.1892*** | 0.1345*** | 0.1650*** | 0.0031 | 0.1772*** | 0.2321*** | 0.1257*** | 0.1526*** | 0.1780*** | -0.0015 | 0.1550*** | 0.2230*** |
| | (0.0339) | (0.0460) | (0.0379) | (0.0559) | (0.0516) | (0.0363) | (0.0871) | (0.0293) | (0.0552) | (0.0516) | (0.0150) | (0.0605) | (0.0505) | (0.0328) | (0.0338) | (0.0303) | (0.0290) | (0.0490) | (0.0275) | (0.0203) | (0.0448) | (0.0293) | (0.0451) | (0.0361) | (0.0382) |
| Girl child_3 | 0.0269 | 0.2210*** | -0.0003 | 0.1267*** | | | 0.2104*** | 0.1745*** | 0.0824*** | 0.2576*** | | 0.2793*** | 0.2157*** | 0.2282*** | 0.1467*** | 0.2041*** | 0.0262 | 0.1607*** | 0.1954*** | 0.0515** | 0.2126*** | 0.1847*** | -0.0413 | 0.1617*** | 0.2722*** |
| | (0.0315) | (0.0386) | (0.0430) | (0.0440) | | | (0.0497) | (0.0302) | (0.0313) | (0.0482) | | (0.0470) | (0.0467) | (0.0352) | (0.0358) | (0.0309) | (0.0316) | (0.0434) | (0.0225) | (0.0219) | (0.0391) | (0.0325) | (0.0405) | (0.0587) | (0.0471) |
| Girl child_4 | -0.0368 | 0.1681*** | 0.0647 | | | | | 0.1759*** | | 0.0953 | | 0.1881*** | | 0.2413*** | 0.1143** | | 0.0733 | | | 0.0874*** | | | | 0.2352*** | 0.1946*** |
| | (0.0334) | (0.0342) | (0.0413) | | | | | (0.0367) | | (0.0596) | | (0.0427) | | (0.0416) | (0.0497) | | (0.0489) | | | (0.0194) | | | | (0.0630) | (0.0291) |
| Girl child_5 | 0.0117 | | | | | | | 0.1588*** | | 0.1152*** | | 0.2044*** | | | | | | | | 0.0813*** | | | | | |
| | (0.0301) | | | | | | | (0.0337) | | (0.0445) | | (0.0284) | | | | | | | | (0.0200) | | | | | |
| Girl child_6 | | | | | | | | | | | | | | | | | | | | 0.0412** | | | | | |
| | | | | | | | | | | | | | | | | | | | | (0.0188) | | | | | |
| Girl child_7 | | | | | | | | | | | | | | | | | | | | 0.0452** | | | | | |
| | | | | | | | | | | | | | | | | | | | | (0.0189) | | | | | |
| Firstborn_1 | 0.0887 | -0.0499 | 0.1337 | -0.1190 | 0.0488 | 0.1262 | -0.0631 | 0.0654 | 0.1345*** | -0.1478 | 0.0463 | 0.1359** | 0.0060 | -0.1240* | -0.0841 | -0.0269 | 0.0925 | -0.1000 | -0.0459 | 0.1355*** | 0.1015* | -0.0292 | 0.1338** | -0.0686 | -0.0509 |
| | (0.0552) | (0.0675) | (0.0842) | (0.1125) | (0.0659) | (0.0986) | (0.0798) | (0.0423) | (0.0484) | (0.0930) | (0.0286) | (0.0640) | (0.0723) | (0.0683) | (0.0758) | (0.0843) | (0.0577) | (0.0683) | (0.0811) | (0.0387) | (0.0593) | (0.0547) | (0.0578) | (0.0638) | (0.0543) |
| Firstborn_2 | -0.1034** | -0.1530* | 0.1168** | 0.0721 | 0.0698 | 0.0751 | 0.0411 | 0.0254 | 0.1819** | -0.1197 | 0.1286*** | 0.1356 | -0.0375 | -0.1315*** | 0.0138 | -0.0887* | 0.0027 | -0.0733 | 0.0928** | 0.1251*** | 0.0613 | -0.0680 | 0.1175** | 0.0409 | -0.1035* |
| | (0.0422) | (0.0843) | (0.0557) | (0.0833) | (0.0802) | (0.0610) | (0.1123) | (0.0386) | (0.0898) | (0.0850) | (0.0195) | (0.0884) | (0.0697) | (0.0483) | (0.0539) | (0.0456) | (0.0382) | (0.0765) | (0.0451) | (0.0279) | (0.0745) | (0.0440) | (0.0581) | (0.0553) | (0.0568) |
| Firstborn_3 | -0.0651 | -0.1186** | -0.0037 | 0.1856*** | | | -0.0882 | 0.0825** | 0.1483*** | 0.0792 | | 0.0617 | 0.1059 | -0.0613 | -0.0819 | -0.1359*** | 0.0694 | 0.0814 | -0.0156 | 0.1460*** | 0.0915* | 0.0729 | 0.1446** | -0.1236 | 0.0113 |
| | (0.0472) | (0.0584) | (0.0565) | (0.0620) | | | (0.0768) | (0.0394) | (0.0448) | (0.0755) | | (0.0629) | (0.0735) | (0.0514) | (0.0533) | (0.0466) | (0.0423) | (0.0752) | (0.0335) | (0.0304) | (0.0534) | (0.0478) | (0.0570) | (0.0948) | (0.0695) |
| Firstborn_4 | -0.0513 | -0.0409 | -0.0180 | | | | | 0.1343*** | | -0.0078 | | 0.1261** | | -0.1605** | 0.1316 | | 0.0619 | | | 0.1271*** | | | | -0.0383 | 0.0398 |
| | (0.0489) | (0.0556) | (0.0554) | | | | | (0.0490) | | (0.0955) | | (0.0607) | | (0.0702) | (0.0868) | | (0.0674) | | | (0.0291) | | | | (0.0952) | (0.0457) |
| Firstborn_5 | -0.0506 | | | | | | | 0.0858** | | -0.1360** | | 0.0104 | | | | | | | | 0.1102*** | | | | | |
| | (0.0411) | | | | | | | (0.0414) | | (0.0640) | | (0.0400) | | | | | | | | (0.0287) | | | | | |
| Firstborn_6 | | | | | | | | | | | | | | | | | | | | 0.1606*** | | | | | |
| | | | | | | | | | | | | | | | | | | | | (0.0268) | | | | | |
| Firstborn_7 | | | | | | | | | | | | | | | | | | | | 0.1336*** | | | | | |
| | | | | | | | | | | | | | | | | | | | | (0.0253) | | | | | |

| | | | | | | | | | | | | | | | | | | | | | | | | | |
|---|---|---|---|---|---|---|---|---|---|---|---|---|---|---|---|---|---|---|---|---|---|---|---|---|---|
| ***Parent's characteristics*** | | | | | | | | | | | | | | | | | | | | | | | | | |
| Mother's height (in 10 cm)_1 | 0.5147*** | 0.4503*** | 0.4260*** | 0.4919*** | 0.4169*** | 0.4034*** | 0.5959*** | 0.3733*** | 0.4461*** | 0.4607*** | 0.5008*** | 0.4741*** | 0.5538*** | 0.4767*** | 0.4401*** | 0.4722*** | 0.5334*** | 0.5027*** | 0.3617*** | 0.6321*** | 0.4731*** | 0.5823*** | 0.5817*** | 0.5022*** | 0.5290*** |
| | (0.0511) | (0.0374) | (0.0535) | (0.0603) | (0.0418) | (0.0622) | (0.0399) | (0.0297) | (0.0311) | (0.0526) | (0.0199) | (0.0386) | (0.0477) | (0.0464) | (0.0375) | (0.0464) | (0.0441) | (0.0403) | (0.0459) | (0.0283) | (0.0373) | (0.0337) | (0.0461) | (0.0368) | (0.0362) |
| Mother's height (in 10 cm)_2 | 0.5519*** | 0.4730*** | 0.5607*** | 0.5589*** | 0.3631*** | 0.4481*** | 0.3169*** | 0.2912*** | 0.4025*** | 0.5233*** | 0.5230*** | 0.3929*** | 0.5001*** | 0.4378*** | 0.4441*** | 0.5368*** | 0.5171*** | 0.4908*** | 0.3084*** | 0.5956*** | 0.5304*** | 0.4880*** | 0.6019*** | 0.5511*** | 0.5555*** |
| | (0.0354) | (0.0463) | (0.0438) | (0.0479) | (0.0477) | (0.0322) | (0.0666) | (0.0318) | (0.0496) | (0.0503) | (0.0151) | (0.0513) | (0.0472) | (0.0308) | (0.0327) | (0.0291) | (0.0329) | (0.0474) | (0.0274) | (0.0233) | (0.0421) | (0.0285) | (0.0470) | (0.0370) | (0.0310) |
| Mother's height (in 10 cm)_3 | 0.6128*** | 0.4475*** | 0.4965*** | 0.4790*** | | | 0.5553*** | 0.4011*** | 0.4786*** | 0.5084*** | | 0.4757*** | 0.4086*** | 0.3651*** | 0.4012*** | 0.4633*** | 0.5493*** | 0.4591*** | 0.4013*** | 0.6034*** | 0.5087*** | 0.5374*** | 0.5678*** | 0.5204*** | 0.4557*** |
| | (0.0319) | (0.0331) | (0.0565) | (0.0389) | | | (0.0496) | (0.0305) | (0.0284) | (0.0457) | | (0.0391) | (0.0476) | (0.0320) | (0.0302) | (0.0307) | (0.0320) | (0.0436) | (0.0223) | (0.0244) | (0.0380) | (0.0292) | (0.0384) | (0.0552) | (0.0398) |
| Mother's height (in 10 cm)_4 | 0.6499*** | 0.4252*** | 0.5374*** | | | | | 0.2874*** | | 0.4560*** | | 0.4475*** | | 0.5223*** | 0.3308*** | | 0.5238*** | | | 0.5955*** | | | | 0.5366*** | 0.4342*** |
| | (0.0348) | (0.0310) | (0.0414) | | | | | (0.0417) | | (0.0502) | | (0.0384) | | (0.0429) | (0.0450) | | (0.0565) | | | (0.0202) | | | | (0.0601) | (0.0258) |
| Mother's height (in 10 cm)_5 | 0.6088*** | | | | | | | 0.4044*** | | 0.5108*** | | 0.4469*** | | | | | | | | 0.6276*** | | | | | |
| | (0.0296) | | | | | | | (0.0337) | | (0.0416) | | (0.0241) | | | | | | | | (0.0203) | | | | | |
| Mother's height (in 10 cm)_6 | | | | | | | | | | | | | | | | | | | | 0.6280*** | | | | | |
| | | | | | | | | | | | | | | | | | | | | (0.0201) | | | | | |
| Mother's height (in 10 cm)_7 | | | | | | | | | | | | | | | | | | | | 0.6355*** | | | | | |
| | | | | | | | | | | | | | | | | | | | | (0.0189) | | | | | |
| Mother's age at birth_1 | 0.0298*** | 0.0315*** | 0.0335*** | 0.0256** | 0.0089 | 0.0278*** | 0.0200*** | 0.0100* | 0.0126*** | 0.0363*** | 0.0253*** | 0.0365*** | 0.0107 | 0.0159* | 0.0196*** | 0.0317*** | 0.0136** | 0.0146** | 0.0199*** | 0.0448*** | 0.0104 | 0.0180*** | 0.0706*** | 0.0217*** | 0.0149** |
| | (0.0061) | (0.0073) | (0.0086) | (0.0104) | (0.0080) | (0.0095) | (0.0064) | (0.0053) | (0.0046) | (0.0113) | (0.0038) | (0.0076) | (0.0084) | (0.0085) | (0.0069) | (0.0084) | (0.0058) | (0.0070) | (0.0071) | (0.0044) | (0.0070) | (0.0066) | (0.0080) | (0.0078) | (0.0066) |
| Mother's age at birth_2 | 0.0373*** | 0.0176** | 0.0248*** | 0.0302*** | -0.0088 | 0.0360*** | 0.0297** | 0.0130*** | 0.0138 | 0.0281*** | 0.0321*** | 0.0318*** | 0.0168** | 0.0132** | 0.0122** | 0.0189*** | 0.0166*** | 0.0240*** | 0.0130*** | 0.0364*** | 0.0322*** | 0.0119** | 0.0562*** | 0.0274*** | 0.0273*** |
| | (0.0055) | (0.0084) | (0.0064) | (0.0094) | (0.0094) | (0.0061) | (0.0121) | (0.0050) | (0.0086) | (0.0088) | (0.0028) | (0.0112) | (0.0066) | (0.0062) | (0.0048) | (0.0045) | (0.0049) | (0.0084) | (0.0045) | (0.0031) | (0.0078) | (0.0052) | (0.0084) | (0.0066) | (0.0072) |
| Mother's age at birth_3 | 0.0333*** | 0.0222*** | 0.0122* | 0.0250*** | | | 0.0242*** | 0.0214*** | -0.0011 | 0.0343*** | | 0.0297*** | 0.0177** | 0.0243*** | 0.0132*** | 0.0227*** | 0.0158** | 0.0261*** | 0.0169*** | 0.0341*** | 0.0201*** | 0.0104* | 0.0392*** | 0.0245** | 0.0255*** |
| | (0.0048) | (0.0058) | (0.0066) | (0.0065) | | | (0.0067) | (0.0051) | (0.0046) | (0.0073) | | (0.0078) | (0.0071) | (0.0065) | (0.0051) | (0.0046) | (0.0063) | (0.0071) | (0.0035) | (0.0035) | (0.0070) | (0.0054) | (0.0076) | (0.0115) | (0.0077) |
| Mother's age at birth_4 | 0.0246*** | 0.0264*** | 0.0185*** | | | | | 0.0183*** | | 0.0247*** | | 0.0096 | | 0.0267*** | 0.0178** | | 0.0234** | | | 0.0303*** | | | | 0.0457*** | 0.0242*** |
| | (0.0055) | (0.0058) | (0.0064) | | | | | (0.0065) | | (0.0077) | | (0.0071) | | (0.0085) | (0.0083) | | (0.0091) | | | (0.0030) | | | | (0.0103) | (0.0046) |
| Mother's age at birth_5 | 0.0276*** | | | | | | | 0.0126** | | 0.0257*** | | 0.0277*** | | | | | | | | 0.0274*** | | | | | |
| | (0.0049) | | | | | | | (0.0053) | | (0.0061) | | (0.0044) | | | | | | | | (0.0027) | | | | | |
| Mother's age at birth_6 | | | | | | | | | | | | | | | | | | | | 0.0271*** | | | | | |
| | | | | | | | | | | | | | | | | | | | | (0.0027) | | | | | |
| Mother's age at birth_7 | | | | | | | | | | | | | | | | | | | | 0.0256*** | | | | | |
| | | | | | | | | | | | | | | | | | | | | (0.0025) | | | | | |
| Mother's age at first marriage_1 | -0.0034 | -0.0108 | -0.0298*** | -0.0055 | 0.0163 | 0.0015 | -0.0043 | -0.0043 | 0.0085 | -0.0289** | 0.0185*** | -0.0045 | 0.0028 | -0.0066 | -0.0108 | -0.0038 | 0.0024 | -0.0108 | 0.0234** | -0.0134*** | 0.0137 | -0.0174** | -0.0289*** | 0.0075 | -0.0006 |
| | (0.0091) | (0.0126) | (0.0100) | (0.0151) | (0.0103) | (0.0118) | (0.0076) | (0.0064) | (0.0064) | (0.0145) | (0.0049) | (0.0088) | (0.0106) | (0.0113) | (0.0110) | (0.0101) | (0.0072) | (0.0124) | (0.0092) | (0.0052) | (0.0088) | (0.0075) | (0.0096) | (0.0093) | (0.0090) |
| Mother's age at first marriage_2 | 0.0057 | 0.0194 | -0.0166** | -0.0119 | 0.0102 | -0.0181** | 0.0010 | -0.0035 | -0.0037 | -0.0081 | 0.0036 | 0.0019 | 0.0036 | -0.0108 | 0.0027 | -0.0090 | -0.0136* | 0.0197* | 0.0044 | -0.0105*** | -0.0237** | -0.0122** | -0.0162* | 0.0001 | -0.0169* |
| | (0.0078) | (0.0124) | (0.0082) | (0.0107) | (0.0113) | (0.0071) | (0.0133) | (0.0064) | (0.0099) | (0.0107) | (0.0034) | (0.0126) | (0.0093) | (0.0074) | (0.0064) | (0.0060) | (0.0082) | (0.0106) | (0.0055) | (0.0037) | (0.0107) | (0.0062) | (0.0093) | (0.0080) | (0.0087) |
| Mother's age at first marriage_3 | -0.0038 | -0.0115 | -0.0053 | 0.0128 | | | -0.0168** | -0.0146** | 0.0012 | -0.0263*** | | 0.0032 | 0.0004 | -0.0292*** | -0.0036 | 0.0009 | 0.0024 | -0.0093 | 0.0123*** | -0.0036 | -0.0115 | -0.0144** | -0.0154* | -0.0161 | -0.0061 |
| | (0.0086) | (0.0088) | (0.0080) | (0.0080) | | | (0.0079) | (0.0062) | (0.0064) | (0.0084) | | (0.0096) | (0.0101) | (0.0082) | (0.0069) | (0.0049) | (0.0080) | (0.0107) | (0.0042) | (0.0041) | (0.0097) | (0.0068) | (0.0091) | (0.0131) | (0.0093) |
| Mother's age at first marriage_4 | -0.0116 | -0.0150* | -0.0072 | | | | | -0.0173** | | -0.0104 | | -0.0022 | | -0.0347*** | -0.0151 | | -0.0103 | | | -0.0115*** | | | | -0.0026 | -0.0025 |
| | (0.0090) | (0.0088) | (0.0070) | | | | | (0.0081) | | (0.0107) | | (0.0088) | | (0.0102) | (0.0097) | | (0.0115) | | | (0.0038) | | | | (0.0117) | (0.0058) |
| Mother's age at first marriage_5 | 0.0167** | | | | | | | -0.0020 | | -0.0003 | | -0.0170*** | | | | | | | | -0.0101*** | | | | | |
| | (0.0071) | | | | | | | (0.0065) | | (0.0071) | | (0.0056) | | | | | | | | (0.0035) | | | | | |
| Mother's age at first marriage_6 | | | | | | | | | | | | | | | | | | | | -0.0097*** | | | | | |
| | | | | | | | | | | | | | | | | | | | | (0.0032) | | | | | |
| Mother's age at first marriage_7 | | | | | | | | | | | | | | | | | | | | -0.0040 | | | | | |
| | | | | | | | | | | | | | | | | | | | | (0.0031) | | | | | |
| Mother's fertility_1 | -0.0865*** | -0.0627*** | -0.0500** | -0.0674** | 0.0042 | -0.0369 | -0.0196 | -0.0132 | -0.0251* | -0.0936*** | -0.0699*** | -0.0531*** | -0.0518** | -0.0241 | -0.0398** | -0.0404* | -0.0389** | -0.0466** | 0.0015 | -0.1130*** | -0.0531*** | -0.0379** | -0.1209*** | -0.0341* | -0.0226 |
| | (0.0195) | (0.0209) | (0.0249) | (0.0285) | (0.0204) | (0.0275) | (0.0170) | (0.0135) | (0.0140) | (0.0348) | (0.0112) | (0.0187) | (0.0212) | (0.0229) | (0.0173) | (0.0230) | (0.0175) | (0.0184) | (0.0192) | (0.0120) | (0.0200) | (0.0190) | (0.0203) | (0.0197) | (0.0188) |
| Mother's fertility_2 | -0.1191*** | -0.0443** | -0.0737*** | -0.0648** | 0.0338 | -0.0827*** | -0.0308 | -0.0253* | 0.0246 | -0.0875*** | -0.0802*** | -0.0163 | -0.0446** | -0.0341** | -0.0113 | -0.0441*** | -0.0577*** | -0.0601*** | -0.0144 | -0.1070*** | -0.0795*** | -0.0379*** | -0.0863*** | -0.0635*** | -0.0776*** |
| | (0.0177) | (0.0223) | (0.0187) | (0.0262) | (0.0234) | (0.0163) | (0.0370) | (0.0150) | (0.0279) | (0.0257) | (0.0085) | (0.0295) | (0.0204) | (0.0173) | (0.0129) | (0.0131) | (0.0162) | (0.0214) | (0.0123) | (0.0089) | (0.0249) | (0.0135) | (0.0237) | (0.0175) | (0.0204) |
| Mother's fertility_3 | -0.1394*** | -0.0484*** | -0.0567** | -0.0295 | | | -0.0525*** | -0.0540*** | 0.0177 | -0.0944*** | | -0.0648*** | -0.0645*** | -0.0656*** | -0.0256* | -0.0654*** | -0.0630*** | -0.0456** | -0.0341*** | -0.0947*** | -0.0992*** | -0.0267* | -0.0514** | -0.0621** | -0.0453** |
| | (0.0163) | (0.0162) | (0.0225) | (0.0198) | | | (0.0196) | (0.0164) | (0.0138) | (0.0216) | | (0.0228) | (0.0210) | (0.0217) | (0.0137) | (0.0143) | (0.0187) | (0.0186) | (0.0099) | (0.0107) | (0.0227) | (0.0159) | (0.0227) | (0.0301) | (0.0229) |
| Mother's fertility_4 | -0.1215*** | -0.0566*** | -0.0824*** | | | | | -0.0395* | | -0.0341 | | -0.0004 | | -0.0823*** | -0.0274 | | -0.0903*** | | | -0.1184*** | | | | -0.0854*** | -0.0488*** |
| | (0.0201) | (0.0176) | (0.0237) | | | | | (0.0229) | | (0.0290) | | (0.0231) | | (0.0266) | (0.0210) | | (0.0304) | | | (0.0102) | | | | (0.0292) | (0.0141) |
| Mother's fertility_5 | -0.0950*** | | | | | | | -0.0265 | | -0.1018*** | | -0.0767*** | | | | | | | | -0.1168*** | | | | | |
| | (0.0195) | | | | | | | (0.0211) | | (0.0216) | | (0.0144) | | | | | | | | (0.0100) | | | | | |
| Mother's fertility_6 | | | | | | | | | | | | | | | | | | | | -0.0931*** | | | | | |
| | | | | | | | | | | | | | | | | | | | | (0.0102) | | | | | |
| Mother's fertility_7 | | | | | | | | | | | | | | | | | | | | -0.0863*** | | | | | |
| | | | | | | | | | | | | | | | | | | | | (0.0093) | | | | | |
| Mother has no education_1 | -0.1868*** | -0.1731** | -0.1666** | -0.4067*** | -0.2445*** | 0.0035 | -0.1237** | 0.0046 | -0.1112** | -0.0373 | -0.3470*** | -0.0535 | -0.0298 | -0.1317** | -0.0238 | -0.1441** | -0.2510*** | -0.0717 | -0.3376*** | -0.2505*** | -0.0675 | -0.0491 | -0.3469*** | -0.0204 | -0.0632 |
| | (0.0529) | (0.0686) | (0.0680) | (0.1284) | (0.0673) | (0.0999) | (0.0562) | (0.0381) | (0.0498) | (0.0830) | (0.0256) | (0.0720) | (0.0740) | (0.0627) | (0.0702) | (0.0678) | (0.0603) | (0.0748) | (0.0751) | (0.0543) | (0.0491) | (0.0491) | (0.0628) | (0.0591) | (0.0609) |
| Mother has no education_2 | -0.1265*** | -0.0962 | -0.1226** | -0.2277** | -0.1703** | -0.0731 | -0.1671* | -0.0664* | -0.1821** | -0.0214 | -0.2070*** | -0.2574** | 0.0642 | -0.0938** | -0.1591*** | -0.1359*** | -0.1602*** | -0.1238* | -0.2318*** | -0.0658 | -0.0405 | -0.0497 | -0.4767*** | -0.1479*** | -0.0224 |
| | (0.0424) | (0.0967) | (0.0581) | (0.0987) | (0.0783) | (0.0526) | (0.0978) | (0.0383) | (0.0764) | (0.0652) | (0.0220) | (0.1158) | (0.0699) | (0.0414) | (0.0506) | (0.0380) | (0.0396) | (0.0709) | (0.0461) | (0.0471) | (0.0576) | (0.0402) | (0.0761) | (0.0558) | (0.0682) |
| Mother has no education_3 | -0.0405 | -0.2468*** | -0.1343** | -0.2110*** | | | -0.1130* | -0.0526 | -0.1148*** | -0.1537** | | 0.0090 | 0.1359* | -0.0668 | -0.1573*** | -0.1075*** | -0.1909*** | -0.1226* | -0.2575*** | -0.0697 | -0.1041* | -0.0582 | -0.2762*** | 0.0363 | 0.1395 |
| | (0.0402) | (0.0600) | (0.0621) | (0.0811) | | | (0.0626) | (0.0398) | (0.0442) | (0.0704) | | (0.0926) | (0.0727) | (0.0493) | (0.0558) | (0.0398) | (0.0447) | (0.0729) | (0.0400) | (0.0507) | (0.0570) | (0.0434) | (0.0839) | (0.0855) | (0.0863) |
| Mother has no education_4 | 0.0214 | -0.1279** | 0.1231* | | | | | -0.0028 | | -0.0388 | | 0.1189 | | -0.0706 | -0.0427 | | -0.1559** | | | -0.1929*** | | | | -0.2836** | -0.0542 |
| | (0.0470) | (0.0546) | (0.0648) | | | | | (0.0537) | | (0.0781) | | (0.0874) | | (0.0682) | (0.0789) | | (0.0664) | | | (0.0564) | | | | (0.1214) | (0.0513) |
| Mother has no education_5 | -0.0935* | | | | | | | -0.0531 | | -0.0718 | | 0.1853*** | | | | | | | | -0.1428** | | | | | |
| | (0.0478) | | | | | | | (0.0518) | | (0.0667) | | (0.0609) | | | | | | | | (0.0599) | | | | | |
| Mother has no education_6 | | | | | | | | | | | | | | | | | | | | -0.1934*** | | | | | |
| | | | | | | | | | | | | | | | | | | | | (0.0662) | | | | | |
| Mother has no education_7 | | | | | | | | | | | | | | | | | | | | -0.1066* | | | | | |
| | | | | | | | | | | | | | | | | | | | | (0.0607) | | | | | |
| Father has no education_1 | -0.1988*** | -0.1852** | -0.0482 | -0.1047 | -0.1584** | 0.0103 | -0.0136 | -0.1032** | -0.1322*** | 0.0039 | -0.1216*** | -0.0632 | 0.0947 | -0.0184 | -0.1537** | -0.0952 | -0.1434*** | -0.1780** | -0.2805*** | -0.0193 | -0.1363*** | -0.1404** | -0.3573*** | 0.0402 | 0.0874 |
| | (0.0510) | (0.0769) | (0.0809) | (0.1248) | (0.0655) | (0.1237) | (0.0570) | (0.0447) | (0.0430) | (0.1051) | (0.0287) | (0.1005) | (0.0813) | (0.0800) | (0.0693) | (0.0902) | (0.0474) | (0.0757) | (0.0740) | (0.1113) | (0.0478) | (0.0601) | (0.1341) | (0.0879) | (0.0866) |
| Father has no education_2 | -0.1494*** | -0.0976 | -0.1316** | -0.0537 | -0.3069*** | -0.1381* | -0.2947 | -0.0791* | -0.0968 | -0.1443* | -0.1164*** | -0.1888 | -0.0265 | -0.1445*** | -0.1906*** | -0.0857* | -0.0333 | -0.1412* | -0.0203 | -0.2062*** | -0.0981 | -0.0969** | -0.5645*** | -0.1283 | -0.0475 |
| | (0.0445) | (0.0910) | (0.0662) | (0.1018) | (0.0703) | (0.0780) | (0.1854) | (0.0456) | (0.0725) | (0.0813) | (0.0241) | (0.1434) | (0.0914) | (0.0541) | (0.0506) | (0.0484) | (0.0445) | (0.0760) | (0.0451) | (0.0767) | (0.0612) | (0.0494) | (0.1256) | (0.0863) | (0.0861) |
| Father has no education_3 | -0.1253*** | -0.1402** | -0.0978 | -0.1414 | | | 0.0448 | 0.0476 | -0.0594 | -0.1355* | | 0.2198* | -0.1187 | -0.0707 | -0.1120** | -0.0903* | -0.0638 | -0.0862 | -0.1397*** | 0.0288 | -0.0491 | 0.0201 | -0.2530** | -0.3882*** | 0.0429 |
| | (0.0402) | (0.0604) | (0.0735) | (0.0883) | | | (0.0655) | (0.0441) | (0.0417) | (0.0729) | | (0.1140) | (0.0806) | (0.0537) | (0.0535) | (0.0499) | (0.0477) | (0.0654) | (0.0408) | (0.0788) | (0.0620) | (0.0514) | (0.1019) | (0.1104) | (0.1183) |
| Father has no education_4 | -0.1645*** | -0.0411 | -0.0635 | | | | | -0.0900 | | -0.0739 | | -0.0734 | | -0.0087 | -0.2076*** | | 0.0135 | | | 0.0250 | | | | 0.0804 | -0.0413 |
| | (0.0456) | (0.0523) | (0.0704) | | | | | (0.0639) | | (0.0879) | | (0.0914) | | (0.0880) | (0.0804) | | (0.0840) | | | (0.1025) | | | | (0.1217) | (0.0712) |
| Father has no education_5 | -0.1088** | | | | | | | -0.0979* | | -0.1433** | | 0.1351** | | | | | | | | 0.0147 | | | | | |
| | (0.0435) | | | | | | | (0.0570) | | (0.0645) | | (0.0647) | | | | | | | | (0.0954) | | | | | |
| Father has no education_6 | | | | | | | | | | | | | | | | | | | | 0.0468 | | | | | |
| | | | | | | | | | | | | | | | | | | | | (0.1104) | | | | | |
| Father has no education_7 | | | | | | | | | | | | | | | | | | | | -0.0417 | | | | | |
| | | | | | | | | | | | | | | | | | | | | (0.0916) | | | | | |

| | | | | | | | | | | | | | | | | | | | | | | | | | |
|---|---|---|---|---|---|---|---|---|---|---|---|---|---|---|---|---|---|---|---|---|---|---|---|---|---|
| ***Household characteristics*** | | | | | | | | | | | | | | | | | | | | | | | | | |
| Household in the first wealth quintile_1 | -0.0030 | -0.0788 | -0.1539** | -0.1230 | -0.0721 | -0.0346 | -0.0192 | 0.0189 | 0.0539 | -0.2473*** | -0.0449 | -0.1344** | -0.0713 | -0.2092*** | -0.0087 | -0.0491 | -0.1055** | -0.0663 | -0.0048 | -0.2189*** | -0.1257*** | -0.1496*** | -0.1927** | -0.1200* | -0.0807 |
| | (0.0562) | (0.0701) | (0.0682) | (0.1136) | (0.0717) | (0.1106) | (0.0752) | (0.0499) | (0.0483) | (0.0909) | (0.0379) | (0.0615) | (0.0668) | (0.0764) | (0.0689) | (0.0888) | (0.0485) | (0.0580) | (0.0908) | (0.0519) | (0.0479) | (0.0525) | (0.0766) | (0.0708) | (0.0517) |
| Household in the first wealth quintile_2 | -0.1681*** | -0.1126 | -0.0920* | -0.0108 | -0.1374 | -0.1065** | -0.2656 | -0.0808 | -0.0721 | -0.1244* | -0.0837*** | -0.2666*** | -0.1290 | -0.1539*** | -0.0813 | -0.0829* | -0.2357*** | 0.0131 | -0.1928*** | -0.1467*** | -0.2168*** | -0.2261*** | -0.2227*** | -0.1106* | -0.1826*** |
| | (0.0518) | (0.0700) | (0.0524) | (0.0935) | (0.1020) | (0.0526) | (0.1918) | (0.0516) | (0.0794) | (0.0755) | (0.0304) | (0.0855) | (0.0841) | (0.0476) | (0.0523) | (0.0496) | (0.0414) | (0.0779) | (0.0488) | (0.0409) | (0.0652) | (0.0445) | (0.0764) | (0.0567) | (0.0546) |
| Household in the first wealth quintile_3 | -0.1979*** | -0.2299*** | -0.1859*** | -0.2496*** | | | -0.1166* | -0.1574*** | -0.0681 | -0.0885 | | -0.2073*** | -0.0923 | -0.2158*** | -0.0893* | -0.1455*** | -0.1227** | 0.0168 | -0.1886*** | -0.1930*** | -0.2256*** | -0.1734*** | -0.3831*** | -0.1712* | -0.0620 |
| | (0.0457) | (0.0561) | (0.0652) | (0.0797) | | | (0.0663) | (0.0454) | (0.0435) | (0.0794) | | (0.0716) | (0.0763) | (0.0517) | (0.0518) | (0.0541) | (0.0478) | (0.0730) | (0.0478) | (0.0385) | (0.0541) | (0.0512) | (0.0630) | (0.0923) | (0.0597) |
| Household in the first wealth quintile_4 | -0.1136* | -0.0243 | -0.0889 | | | | | 0.0023 | | -0.0759 | | -0.1867*** | | -0.2160*** | -0.1269* | | -0.2107** | | | -0.1905*** | | | | -0.1633 | -0.1425*** |
| | (0.0581) | (0.0572) | (0.0586) | | | | | (0.0590) | | (0.1023) | | (0.0674) | | (0.0597) | (0.0754) | | (0.0916) | | | (0.0406) | | | | (0.1029) | (0.0408) |
| Household in the first wealth quintile_5 | -0.2917*** | | | | | | | -0.0823 | | 0.0334 | | -0.3178*** | | | | | | | | -0.3103*** | | | | | |
| | (0.0503) | | | | | | | (0.0501) | | (0.0742) | | (0.0472) | | | | | | | | (0.0363) | | | | | |
| Household in the first wealth quintile_6 | | | | | | | | | | | | | | | | | | | | -0.3066*** | | | | | |
| | | | | | | | | | | | | | | | | | | | | (0.0341) | | | | | |
| Household in the first wealth quintile_7 | | | | | | | | | | | | | | | | | | | | -0.3038*** | | | | | |
| | | | | | | | | | | | | | | | | | | | | (0.0344) | | | | | |
| Rural household_1 | -0.1541** | 0.1114 | 0.0306 | -0.1380 | -0.1666* | -0.3886*** | -0.2428*** | -0.2681*** | 0.1164 | -0.1567 | -0.2945*** | -0.1042 | -0.1100 | -0.4205*** | -0.2274*** | -0.4165*** | -0.1516** | 0.0324 | -0.0608 | -0.0741 | -0.4059*** | -0.3049*** | -0.1500** | -0.4129*** | -0.2924*** |
| | (0.0667) | (0.1473) | (0.0927) | (0.1129) | (0.0953) | (0.1129) | (0.0817) | (0.0588) | (0.1174) | (0.1108) | (0.0362) | (0.0914) | (0.0959) | (0.0866) | (0.0676) | (0.0899) | (0.0770) | (0.1331) | (0.0894) | (0.0620) | (0.0865) | (0.0685) | (0.0669) | (0.0643) | (0.0641) |
| Rural household_2 | -0.1590*** | -0.4758*** | 0.0444 | -0.2472*** | 0.0352 | -0.1773*** | -0.1032 | 0.0184 | -0.6028*** | -0.2476*** | -0.0017 | -0.2202* | -0.0254 | -0.3607*** | -0.4726*** | -0.2782*** | -0.0930 | -0.4973*** | -0.2755*** | -0.1291*** | -0.2205*** | -0.1679*** | -0.1245** | -0.3017*** | -0.2295*** |
| | (0.0602) | (0.1715) | (0.0660) | (0.0868) | (0.1542) | (0.0650) | (0.1433) | (0.0500) | (0.1608) | (0.0837) | (0.0248) | (0.1174) | (0.0900) | (0.0629) | (0.0737) | (0.0500) | (0.0696) | (0.1408) | (0.0616) | (0.0497) | (0.0814) | (0.0614) | (0.0609) | (0.0743) | (0.0702) |
| Rural household_3 | -0.0565 | -0.2161** | -0.0610 | -0.1222 | | | -0.1337* | -0.2558*** | -0.2789*** | -0.1846** | | -0.1515* | -0.1570 | -0.3422*** | -0.3118*** | -0.0575 | -0.1567*** | -0.1018 | -0.2014*** | -0.2692*** | -0.4154*** | -0.2493*** | -0.1339** | -0.4150*** | -0.2649*** |
| | (0.0555) | (0.0971) | (0.0712) | (0.0749) | | | (0.0800) | (0.0644) | (0.0835) | (0.0848) | | (0.0866) | (0.1012) | (0.0793) | (0.0696) | (0.0577) | (0.0584) | (0.1076) | (0.0511) | (0.0429) | (0.0843) | (0.0670) | (0.0563) | (0.1217) | (0.0861) |
| Rural household_4 | -0.1009** | -0.1420* | -0.0593 | | | | | -0.0535 | | -0.1670* | | -0.1483** | | -0.1381* | -0.5421*** | | -0.3289*** | | | -0.3931*** | | | | -0.2361** | 0.0180 |
| | (0.0483) | (0.0784) | (0.0644) | | | | | (0.0707) | | (0.1008) | | (0.0748) | | (0.0815) | (0.1043) | | (0.0774) | | | (0.0366) | | | | (0.1048) | (0.0511) |
| Rural household_5 | -0.1290*** | | | | | | | -0.0258 | | -0.1148** | | -0.0309 | | | | | | | | -0.2828*** | | | | | |
| | (0.0431) | | | | | | | (0.0625) | | (0.0580) | | (0.0414) | | | | | | | | (0.0377) | | | | | |
| Rural household_6 | | | | | | | | | | | | | | | | | | | | -0.3388*** | | | | | |
| | | | | | | | | | | | | | | | | | | | | (0.0355) | | | | | |
| Rural household_7 | | | | | | | | | | | | | | | | | | | | -0.2643*** | | | | | |
| | | | | | | | | | | | | | | | | | | | | (0.0333) | | | | | |
| Household size_1 | 0.0095 | -0.0082 | -0.0260 | -0.0044 | 0.0002 | 0.0061 | -0.0043 | -0.0073* | 0.0104 | -0.0005 | -0.0120*** | -0.0059 | 0.0081 | -0.0099 | -0.0165*** | 0.0028 | 0.0033 | -0.0033 | -0.0295*** | -0.0244*** | 0.0709*** | -0.0053 | -0.0302*** | -0.0090 | 0.0006 |
| | (0.0067) | (0.0050) | (0.0188) | (0.0094) | (0.0063) | (0.0125) | (0.0034) | (0.0044) | (0.0101) | (0.0173) | (0.0031) | (0.0066) | (0.0135) | (0.0113) | (0.0060) | (0.0115) | (0.0066) | (0.0047) | (0.0084) | (0.0056) | (0.0133) | (0.0067) | (0.0083) | (0.0082) | (0.0068) |
| Household size_2 | -0.0055 | 0.0062 | -0.0035 | -0.0159** | -0.0108 | -0.0094 | -0.0018 | -0.0038 | -0.0286 | -0.0031 | -0.0015 | -0.0242 | -0.0182* | -0.0032 | -0.0116** | -0.0136*** | -0.0045 | -0.0021 | -0.0086 | -0.0179*** | -0.0011 | 0.0062 | 0.0002 | 0.0111 | -0.0163** |
| | (0.0059) | (0.0045) | (0.0109) | (0.0077) | (0.0080) | (0.0081) | (0.0067) | (0.0053) | (0.0191) | (0.0142) | (0.0028) | (0.0159) | (0.0110) | (0.0084) | (0.0056) | (0.0048) | (0.0064) | (0.0061) | (0.0053) | (0.0047) | (0.0191) | (0.0046) | (0.0089) | (0.0097) | (0.0074) |
| Household size_3 | 0.0016 | -0.0048 | -0.0132 | 0.0070 | | | -0.0150** | -0.0016 | 0.0138 | 0.0113 | | 0.0037 | -0.0106 | 0.0142 | -0.0121** | 0.0119 | -0.0001 | -0.0058 | -0.0100** | -0.0167*** | 0.0303* | 0.0003 | -0.0348*** | 0.0080 | -0.0000 |
| | (0.0058) | (0.0041) | (0.0115) | (0.0069) | | | (0.0063) | (0.0051) | (0.0095) | (0.0110) | | (0.0128) | (0.0116) | (0.0106) | (0.0051) | (0.0074) | (0.0064) | (0.0067) | (0.0044) | (0.0058) | (0.0164) | (0.0048) | (0.0099) | (0.0137) | (0.0103) |
| Household size_4 | 0.0100 | -0.0085 | -0.0002 | | | | | 0.0052 | | -0.0132 | | -0.0329*** | | 0.0029 | -0.0047 | | 0.0107 | | | -0.0165*** | | | | 0.0045 | -0.0034 |
| | (0.0074) | (0.0058) | (0.0092) | | | | | (0.0078) | | (0.0166) | | (0.0110) | | (0.0117) | (0.0095) | | (0.0107) | | | (0.0051) | | | | (0.0160) | (0.0067) |
| Household size_5 | -0.0043 | | | | | | | 0.0094 | | -0.0197** | | -0.0051 | | | | | | | | -0.0145*** | | | | | |
| | (0.0061) | | | | | | | (0.0091) | | (0.0097) | | (0.0091) | | | | | | | | (0.0056) | | | | | |
| Household size_6 | | | | | | | | | | | | | | | | | | | | -0.0065 | | | | | |
| | | | | | | | | | | | | | | | | | | | | (0.0060) | | | | | |
| Household size_7 | | | | | | | | | | | | | | | | | | | | -0.0093 | | | | | |
| | | | | | | | | | | | | | | | | | | | | (0.0057) | | | | | |
| Household has electricity_1 | 0.3983*** | 0.3481*** | -0.0481 | 0.1420 | 0.2532** | 0.4147*** | 0.2506*** | 0.1665** | 0.6726*** | 0.2085* | 0.0995*** | 0.5924*** | 0.4250*** | 0.5019*** | 0.1261 | 0.2677** | 0.1061 | 0.3475*** | 0.3125*** | 0.1506*** | 0.4834*** | 0.3252*** | | 0.2918*** | 0.3844*** |
| | (0.0587) | (0.0836) | (0.1154) | (0.1179) | (0.1123) | (0.1443) | (0.0678) | (0.0731) | (0.1061) | (0.1144) | (0.0318) | (0.0993) | (0.1110) | (0.1100) | (0.0944) | (0.1173) | (0.0727) | (0.1119) | (0.0863) | (0.0506) | (0.1078) | (0.0898) | | (0.0898) | (0.0735) |
| Household has electricity_2 | 0.2903*** | 0.4129*** | 0.1433* | 0.3077*** | 0.2688*** | 0.4931*** | 0.1709 | 0.2311* | 0.2644* | 0.2372*** | 0.1224*** | 0.6487*** | 0.3045*** | 0.4386*** | 0.2366*** | 0.3652*** | 0.3182*** | 0.2449** | 0.0743 | 0.1585*** | 0.9729*** | 0.5811*** | | 0.4753*** | 0.4373*** |
| | (0.0499) | (0.1203) | (0.0751) | (0.0844) | (0.0921) | (0.0946) | (0.1577) | (0.1206) | (0.1418) | (0.0827) | (0.0255) | (0.1300) | (0.0763) | (0.0880) | (0.0714) | (0.0538) | (0.0577) | (0.1018) | (0.0524) | (0.0396) | (0.1460) | (0.0715) | | (0.0788) | (0.0850) |
| Household has electricity_3 | 0.2580*** | 0.3173*** | 0.0672 | 0.2824*** | | | 0.1650** | 0.1011 | 0.3592*** | 0.1881** | | 0.4147*** | 0.2517** | 0.2010** | 0.1289* | 0.3015*** | 0.1260** | -0.1046 | 0.1568*** | 0.1276*** | 0.3048*** | 0.3757*** | | 0.3067* | 0.3501*** |
| | (0.0427) | (0.0962) | (0.0702) | (0.0792) | | | (0.0706) | (0.1708) | (0.0769) | (0.0795) | | (0.1047) | (0.1040) | (0.0977) | (0.0676) | (0.0512) | (0.0581) | (0.0881) | (0.0418) | (0.0370) | (0.0993) | (0.0680) | | (0.1648) | (0.0908) |
| Household has electricity_4 | 0.1925*** | 0.1668* | 0.0549 | | | | | -0.0587 | | 0.1207 | | 0.3345*** | | 0.1092 | -0.0433 | | 0.1303 | | | 0.0585* | | | | 0.3563*** | 0.2432*** |
| | (0.0485) | (0.0855) | (0.0586) | | | | | (0.2165) | | (0.0834) | | (0.0809) | | (0.1017) | (0.1007) | | (0.0892) | | | (0.0348) | | | | (0.1025) | (0.0517) |
| Household has electricity_5 | 0.1086*** | | | | | | | -0.1365 | | 0.0506 | | 0.3737*** | | | | | | | | -0.0593 | | | | | |
| | (0.0417) | | | | | | | (0.3915) | | (0.0612) | | (0.0464) | | | | | | | | (0.0390) | | | | | |
| Household has electricity_6 | | | | | | | | | | | | | | | | | | | | -0.0428 | | | | | |
| | | | | | | | | | | | | | | | | | | | | (0.0360) | | | | | |
| Household has electricity_7 | | | | | | | | | | | | | | | | | | | | -0.0328 | | | | | |
| | | | | | | | | | | | | | | | | | | | | (0.0411) | | | | | |
| ***Village characteristics*** | | | | | | | | | | | | | | | | | | | | | | | | | |
| No sanitation (village average)_1 | 0.3029*** | -0.5653*** | -0.4187** | -0.1112 | 0.0092 | 0.2492 | -0.0914 | -0.5703*** | -0.3666*** | -0.4017*** | 0.0930 | -0.2549** | 0.3281*** | 0.0170 | -0.1335 | -0.2443** | -0.2986*** | -0.3069** | -0.0723 | -0.4302*** | 0.1233 | -0.0528 | -0.3892 | -0.0802 | 0.2848*** |
| | (0.0910) | (0.1736) | (0.1672) | (0.2302) | (0.1264) | (0.2231) | (0.1240) | (0.1743) | (0.1022) | (0.1124) | (0.1086) | (0.1119) | (0.1121) | (0.1591) | (0.1104) | (0.1127) | (0.0982) | (0.1552) | (0.1317) | (0.0728) | (0.3718) | (0.1140) | (0.6727) | (0.1521) | (0.0816) |
| No sanitation (village average)_2 | 0.1342 | -0.0496 | -0.6088*** | -0.1761 | -0.5023** | -0.1256 | -0.2360 | -0.5603*** | 0.2190* | -0.2578*** | -0.3480*** | 0.0095 | 0.2266* | -0.0111 | -0.3107*** | -0.0087 | -0.1060 | -0.0784 | -0.1532** | -0.4199*** | -0.6496 | 0.1641* | 0.0032 | -0.0901 | 0.1246 |
| | (0.1113) | (0.1967) | (0.1105) | (0.1975) | (0.1989) | (0.1123) | (0.2172) | (0.2166) | (0.1142) | (0.0962) | (0.0328) | (0.1388) | (0.1243) | (0.1397) | (0.0811) | (0.0680) | (0.0873) | (0.1814) | (0.0685) | (0.0640) | (0.4501) | (0.0890) | (0.3045) | (0.1428) | (0.1035) |
| No sanitation (village average)_3 | -0.0080 | -0.2680** | -0.2854*** | -0.0184 | | | -0.2187** | -0.8819* | -0.0014 | -0.2508** | | 0.0282 | 0.5406*** | -0.1889 | -0.3727*** | -0.0777 | -0.1680* | -0.4494*** | 0.1245* | -0.3104*** | -0.3493 | -0.0230 | -0.8707** | -0.3151** | 0.2534** |
| | (0.1409) | (0.1126) | (0.1038) | (0.2281) | | | (0.1087) | (0.5289) | (0.0748) | (0.0978) | | (0.1082) | (0.1082) | (0.1399) | (0.0980) | (0.0823) | (0.0920) | (0.1283) | (0.0647) | (0.0643) | (0.7836) | (0.0924) | (0.4408) | (0.1509) | (0.1175) |
| No sanitation (village average)_4 | -0.1918 | -0.2166** | -0.4383*** | | | | | 1.8124* | | -0.1192 | | 0.0345 | | -0.0330 | -0.3562** | | -0.2820** | | | -0.2582*** | | | | 0.2541 | -0.0767 |
| | (0.1546) | (0.0926) | (0.0959) | | | | | (0.9625) | | (0.1192) | | (0.1121) | | (0.1799) | (0.1688) | | (0.1180) | | | (0.0668) | | | | (0.2033) | (0.0818) |
| No sanitation (village average)_5 | -0.1476 | | | | | | | 1.7784** | | -0.1726* | | 0.0140 | | | | | | | | -0.2592*** | | | | | |
| | (0.1663) | | | | | | | (0.8990) | | (0.0922) | | (0.0756) | | | | | | | | (0.0645) | | | | | |
| No sanitation (village average)_6 | | | | | | | | | | | | | | | | | | | | -0.1827*** | | | | | |
| | | | | | | | | | | | | | | | | | | | | (0.0632) | | | | | |
| No sanitation (village average)_7 | | | | | | | | | | | | | | | | | | | | -0.2903*** | | | | | |
| | | | | | | | | | | | | | | | | | | | | (0.0725) | | | | | |
| Mortality in children aged < 5 y  in the village_1 | -0.5942* | 0.1384 | 0.0230 | -0.5307 | -1.0161*** | -0.4514 | -0.2075 | -1.3068*** | -0.5957** | -0.5145 | -0.9245* | -0.7162** | -1.1838*** | -0.2104 | -1.2274*** | -0.0008 | -0.7289* | -1.0497*** | -0.4611 | -0.6587** | -0.0749 | -1.0952*** | -0.8483** | -0.0626 | -0.4742 |
| | (0.3609) | (0.4725) | (0.3638) | (0.5504) | (0.3845) | (0.5938) | (0.4521) | (0.3597) | (0.2776) | (0.3641) | (0.4772) | (0.2880) | (0.4301) | (0.3226) | (0.3779) | (0.4042) | (0.4182) | (0.3419) | (0.3952) | (0.2663) | (0.2720) | (0.3210) | (0.3760) | (0.3356) | (0.3309) |
| Mortality in children aged < 5 y in the village_2 | -0.7847** | 0.0682 | 0.1685 | -0.4958 | -0.1376 | -0.5889 | 0.2371 | -0.6517* | -0.1671 | -0.2014 | -0.5681*** | -0.5098 | -0.4211 | -0.1338 | -0.4206 | -0.6844*** | -1.2935*** | -0.5300 | -0.6482* | -0.3011 | -0.2066 | -0.4982 | -0.2700 | 0.2021 | -0.3026 |
| | (0.3671) | (0.4022) | (0.3624) | (0.6153) | (0.5348) | (0.4326) | (0.5573) | (0.3545) | (0.5359) | (0.3386) | (0.1603) | (0.3659) | (0.5535) | (0.2635) | (0.3524) | (0.2272) | (0.4742) | (0.5459) | (0.3823) | (0.2594) | (0.3733) | (0.3122) | (0.4709) | (0.3746) | (0.3271) |
| Mortality in children aged < 5 y in the village_3 | -0.7354** | -0.4677 | -0.2185 | -0.0772 | | | -0.3818 | -0.0202 | -0.1088 | -0.5901 | | 0.0412 | 0.0010 | 0.0522 | -0.6149 | -0.3499 | -1.5262*** | 0.7368 | -1.9535*** | -0.5610** | -0.7282 | 0.1921 | -0.6939 | -0.8359 | -0.2426 |
| | (0.3665) | (0.3836) | (0.4422) | (0.3698) | | | (0.4191) | (0.5183) | (0.3529) | (0.3626) | | (0.2914) | (0.6481) | (0.3453) | (0.4038) | (0.3138) | (0.4914) | (0.5566) | (0.3911) | (0.2311) | (0.4612) | (0.3399) | (0.4668) | (0.5373) | (0.4234) |
| Mortality in children aged < 5 y in the village_4 | -0.9866*** | -0.7367* | 0.2878 | | | | | 1.5618** | | 0.4829 | | -0.2149 | | -0.1235 | -0.8604 | | 0.4799 | | | -0.4826 | | | | -0.6474 | 0.3423 |
| | (0.3071) | (0.3768) | (0.4303) | | | | | (0.6651) | | (0.4101) | | (0.3798) | | (0.3913) | (0.6526) | | (0.6430) | | | (0.2959) | | | | (0.7160) | (0.3438) |
| Mortality in children aged < 5 y in the village_5 | -0.5267* | | | | | | | -0.8551 | | -0.3122 | | 0.6199** | | | | | | | | -0.2146 | | | | | |
| | (0.3130) | | | | | | | (0.5961) | | (0.4547) | | (0.2865) | | | | | | | | (0.2724) | | | | | |
| Mortality in children aged < 5 y in the village_6 | | | | | | | | | | | | | | | | | | | | -0.1751 | | | | | |
| | | | | | | | | | | | | | | | | | | | | (0.2950) | | | | | |
| Mortality in children aged < 5 y in the village_7 | | | | | | | | | | | | | | | | | | | | -0.2422 | | | | | |
| | | | | | | | | | | | | | | | | | | | | (0.2875) | | | | | |
| Observations | 27,415 | 21,838 | 14,954 | 9,443 | 9,716 | 11,047 | 7,813 | 53,742 | 21,524 | 12,421 | 63,277 | 25,901 | 11,988 | 24,454 | 28,528 | 20,023 | 16,744 | 12,169 | 46,996 | 69,300 | 13,612 | 18,493 | 9,907 | 13,661 | 26,836 |
| Number of clusters | 1,976 | 1,411 | 2,231 | 1,216 | 443 | 836 | 736 | 6,187 | 1,658 | 2,007 | 4,143 | 3,389 | 1,153 | 2,132 | 1,515 | 1,596 | 1,047 | 1,082 | 2,138 | 8,619 | 1,392 | 1,304 | 1,556 | 1,344 | 1,672 |

| Country | 1<br>Bangladesh | 2<br>Burkina Faso | 3<br>Cambodia | 4<br>Cameroon | 5<br>Chad | 6<br>Democratic Republic of Congo | 7<br>Côte d'Ivoire | 8<br>Egypt | 9<br>Ethiopia | 10<br>Ghana | 11<br>India | 12<br>Kenya | 13<br>Madagascar | 14<br>Malawi | 15<br>Mali | 16<br>Mozambique | 17<br>Nepal | 18<br>Niger | 19<br>Nigeria | 20<br>Peru | 21<br>Rwanda | 22<br>Tanzania | 23<br>Turkey | 24<br>Uganda | 25<br>Zambia |
|---|---|---|---|---|---|---|---|---|---|---|---|---|---|---|---|---|---|---|---|---|---|---|---|---|---|
| ***2. Test of equality of coefficients between the first and the last survey round*** | | | | | | | | | | | | | | | | | | | | | | | | | |
| ***Growth faltering indicators*** | | | | | | | | | | | | | | | | | | | | | | | | | |
| Age (in months) | 0.1543 | 0.0041 | 0.0258 | 0.1524 | 0.0067 | 0.2749 | 0.6037 | 0.0000 | 0.8860 | 0.0836 | 0.5795 | 0.0011 | 0.0116 | 0.5586 | 0.2444 | 0.0018 | 0.0792 | 0.0000 | 0.1102 | 0.0000 | 0.0005 | 0.1673 | 0.1247 | 0.2245 | 0.5421 |
| Older than 21 months | 0.0156 | 0.0106 | 0.0283 | 0.0724 | 0.3999 | 0.4403 | 0.0303 | 0.0000 | 0.0201 | 0.0816 | 0.0000 | 0.0004 | 0.4491 | 0.0261 | 0.0004 | 0.6076 | 0.4769 | 0.1940 | 0.4988 | 0.0000 | 0.3557 | 0.9722 | 0.7324 | 0.0365 | 0.3381 |
| Age (in months) * Older than 21 months | 0.2402 | 0.0087 | 0.0694 | 0.4758 | 0.0217 | 0.5017 | 0.0570 | 0.0000 | 0.6185 | 0.1012 | 0.0000 | 0.0008 | 0.2442 | 0.8472 | 0.0062 | 0.7088 | 0.8157 | 0.9640 | 0.0625 | 0.0000 | 0.0156 | 0.1697 | 0.3078 | 0.0587 | 0.8945 |
| ***Child's characteristics*** | | | | | | | | | | | | | | | | | | | | | | | | | |
| Girl child | 0.3957 | 0.6437 | 0.9407 | 0.0393 | 0.3133 | 0.7787 | 0.8415 | 0.2927 | 0.8183 | 0.5413 | 0.1184 | 0.8173 | 0.0488 | 0.8961 | 0.6449 | 0.1011 | 0.5448 | 0.9166 | 0.3844 | 0.0196 | 0.7550 | 0.6608 | 0.4701 | 0.4908 | 0.9234 |
| Firstborn | 0.0430 | 0.9177 | 0.1323 | 0.0178 | 0.8400 | 0.6591 | 0.8206 | 0.7308 | 0.8345 | 0.9166 | 0.0176 | 0.0963 | 0.3330 | 0.7091 | 0.0613 | 0.2579 | 0.7301 | 0.0741 | 0.7301 | 0.9666 | 0.9003 | 0.1598 | 0.8943 | 0.7915 | 0.2009 |
| ***Parent's characteristics*** | | | | | | | | | | | | | | | | | | | | | | | | | |
| Mother's height (in 10 cm) | 0.1110 | 0.6050 | 0.0995 | 0.8570 | 0.3957 | 0.5231 | 0.5244 | 0.4893 | 0.4419 | 0.4553 | 0.3738 | 0.5513 | 0.0312 | 0.4704 | 0.0622 | 0.8721 | 0.8932 | 0.4626 | 0.4376 | 0.9210 | 0.5043 | 0.3146 | 0.8167 | 0.6253 | 0.0331 |
| Mother's age at birth | 0.7752 | 0.5821 | 0.1588 | 0.9650 | 0.1531 | 0.4693 | 0.6494 | 0.7367 | 0.0360 | 0.4056 | 0.1466 | 0.3086 | 0.5218 | 0.3690 | 0.8686 | 0.3441 | 0.3633 | 0.2479 | 0.6993 | 0.0002 | 0.3270 | 0.3734 | 0.0045 | 0.0636 | 0.2489 |
| Mother's age at first marriage | 0.0793 | 0.7815 | 0.0635 | 0.2824 | 0.6885 | 0.1555 | 0.2512 | 0.8017 | 0.4204 | 0.0754 | 0.0122 | 0.2342 | 0.8721 | 0.0639 | 0.7666 | 0.6758 | 0.3480 | 0.9261 | 0.2726 | 0.1228 | 0.0545 | 0.7674 | 0.3054 | 0.5035 | 0.8597 |
| Mother's fertility | 0.7603 | 0.8232 | 0.3457 | 0.2747 | 0.3397 | 0.1524 | 0.2041 | 0.5982 | 0.0296 | 0.8406 | 0.4655 | 0.3172 | 0.6712 | 0.0977 | 0.6489 | 0.3547 | 0.1435 | 0.9699 | 0.0990 | 0.0804 | 0.1274 | 0.6514 | 0.0223 | 0.1449 | 0.2671 |
| Mother has no education | 0.1908 | 0.6067 | 0.0020 | 0.1975 | 0.4720 | 0.4977 | 0.8992 | 0.3690 | 0.9570 | 0.7461 | 0.0000 | 0.0114 | 0.1103 | 0.5094 | 0.8578 | 0.6420 | 0.2892 | 0.6257 | 0.3461 | 0.0773 | 0.6273 | 0.8896 | 0.5004 | 0.0513 | 0.9106 |
| Father has no education | 0.1796 | 0.1211 | 0.8864 | 0.8104 | 0.1223 | 0.3101 | 0.5014 | 0.9416 | 0.2238 | 0.2327 | 0.8910 | 0.0971 | 0.0624 | 0.9350 | 0.6118 | 0.9623 | 0.1037 | 0.3589 | 0.0960 | 0.8765 | 0.2651 | 0.0424 | 0.5357 | 0.7887 | 0.2508 |
| ***Household characteristics*** | | | | | | | | | | | | | | | | | | | | | | | | | |
| Household in the first wealth quintile | 0.0001 | 0.5468 | 0.4698 | 0.3619 | 0.6004 | 0.5573 | 0.3314 | 0.1520 | 0.0605 | 0.0167 | 0.4243 | 0.0180 | 0.8364 | 0.9443 | 0.2470 | 0.3540 | 0.3101 | 0.3729 | 0.0732 | 0.1729 | 0.1665 | 0.7457 | 0.0550 | 0.7293 | 0.3481 |
| Rural household | 0.7515 | 0.1288 | 0.4258 | 0.9069 | 0.2656 | 0.1046 | 0.3398 | 0.0048 | 0.0061 | 0.7375 | 0.0000 | 0.4653 | 0.7357 | 0.0176 | 0.0114 | 0.0008 | 0.1044 | 0.4331 | 0.1720 | 0.0068 | 0.9377 | 0.5617 | 0.8540 | 0.1502 | 0.0002 |
| Household size | 0.1275 | 0.9605 | 0.2183 | 0.3277 | 0.2822 | 0.2970 | 0.1341 | 0.0975 | 0.8034 | 0.3336 | 0.0118 | 0.9444 | 0.2942 | 0.4327 | 0.2966 | 0.5060 | 0.5576 | 0.7658 | 0.0396 | 0.0581 | 0.0539 | 0.4977 | 0.7264 | 0.4550 | 0.6714 |
| Household has electricity | 0.0001 | 0.1295 | 0.4263 | 0.3230 | 0.9142 | 0.6494 | 0.3816 | 0.4469 | 0.0167 | 0.2237 | 0.5755 | 0.0461 | 0.2544 | 0.0088 | 0.2196 | 0.7913 | 0.8331 | 0.0015 | 0.1043 | 0.0049 | 0.2231 | 0.6544 | | 0.6357 | 0.1159 |
| ***Village characteristics*** | | | | | | | | | | | | | | | | | | | | | | | | | |
| No sanitation (village average) | 0.0175 | 0.0764 | 0.9191 | 0.7745 | 0.0299 | 0.1336 | 0.4401 | 0.0103 | 0.0039 | 0.1151 | 0.0001 | 0.0465 | 0.1726 | 0.8351 | 0.2694 | 0.2325 | 0.9138 | 0.4792 | 0.1800 | 0.1732 | 0.5858 | 0.8388 | 0.5493 | 0.1879 | 0.0018 |
| Mortality in children aged < 5 y in the village | 0.8877 | 0.1476 | 0.6384 | 0.4940 | 0.1823 | 0.8515 | 0.7773 | 0.5165 | 0.2782 | 0.7284 | 0.4789 | 0.0010 | 0.1277 | 0.8640 | 0.6264 | 0.4951 | 0.1150 | 0.0062 | 0.0073 | 0.2879 | 0.2225 | 0.0059 | 0.7967 | 0.4596 | 0.0870 |

Panel 1 "Regression results" reports coefficients from a random effects model; the random effect is at cluster/primary sampling unit level.
_1, _2, _3, _4, _5,_6, and _7 indicate an interaction term of the variable with survey round 1, 2, 3, 4, 5, 6, and 7, respectively.
All coefficients capture the association of HAZ and the variable in the corresponding survey round.
Included in the regression but not shown are: a constant; survey round binary variables (1 to 6 dummies for countries with 2 to 7 survey rounds, respectively); indicator that mother has never married, indicator that father's education is missing, and calendar month of birth (set of 11 dummies), all of them are interacted with the survey round binary variables.
Robust standard errors in parentheses
*P < .1; **P < .05; ***P < .01.

Panel 2 "Test of equality of coefficients between the first and the last survey round" reports p-values from the Wald tests.
